# Enhanced retention of porosity in a three-linker glass-forming zeolitic imidazolate framework

**DOI:** 10.1039/d6cc04372k

**Published:** 2026-10-29

**Authors:** Bethan Turner, Harry G. Palmer, Sam D. Harding, Jay McCarron, Chumei Ye, Arad Lang, Louis Longley, Frédéric Blanc, Lauren N. McHugh

**Affiliations:** a Department of Chemistry, University of Liverpool Liverpool L697ZD UK L.N.Mchugh@liverpool.ac.uk; b Materials Innovation Factory, University of Liverpool Liverpool L697ZD UK; c Department of Chemistry, Graduate School of Science, Kyoto University, Kitashirakawa-Oiwakecho, Sakyo-ku Kyoto 606-8502 Japan; d Department of Materials Science and Metallurgy, University of Cambridge Cambridge CB3 0FS UK; e Department of Materials Science and Engineering, Technion – Israel Institute of Technology Haifa 3200003 Israel; f Leverhulme Research Centre for Functional Materials Design University of Liverpool, Liverpool, L697ZD, UK; g Stephenson Institute for Renewable Energy, University of Liverpool Liverpool L697ZF UK

## Abstract

We report a thermally stable, three-linker glass-forming ZIF [Zn(Im)_1.50_(2-mIm)_0.25_(bIm)_0.25_], where crystallographic and nuclear magnetic resonance (NMR) methods confirmed incorporation of an additional linker. CO_2_ adsorption revealed reduced porosity in the crystalline three-linker system, while upon glass formation, an improved retention of porosity was observed.

## Abstract 2

A thermally stable three-linker ZIF, was synthesised and exhibited a high glass-forming ability. The resulting glass demonstrated enhanced retention of porosity and a notable CO_2_ uptake furthering the development of three-linker glass-forming ZIFs.

## Graphical abstract

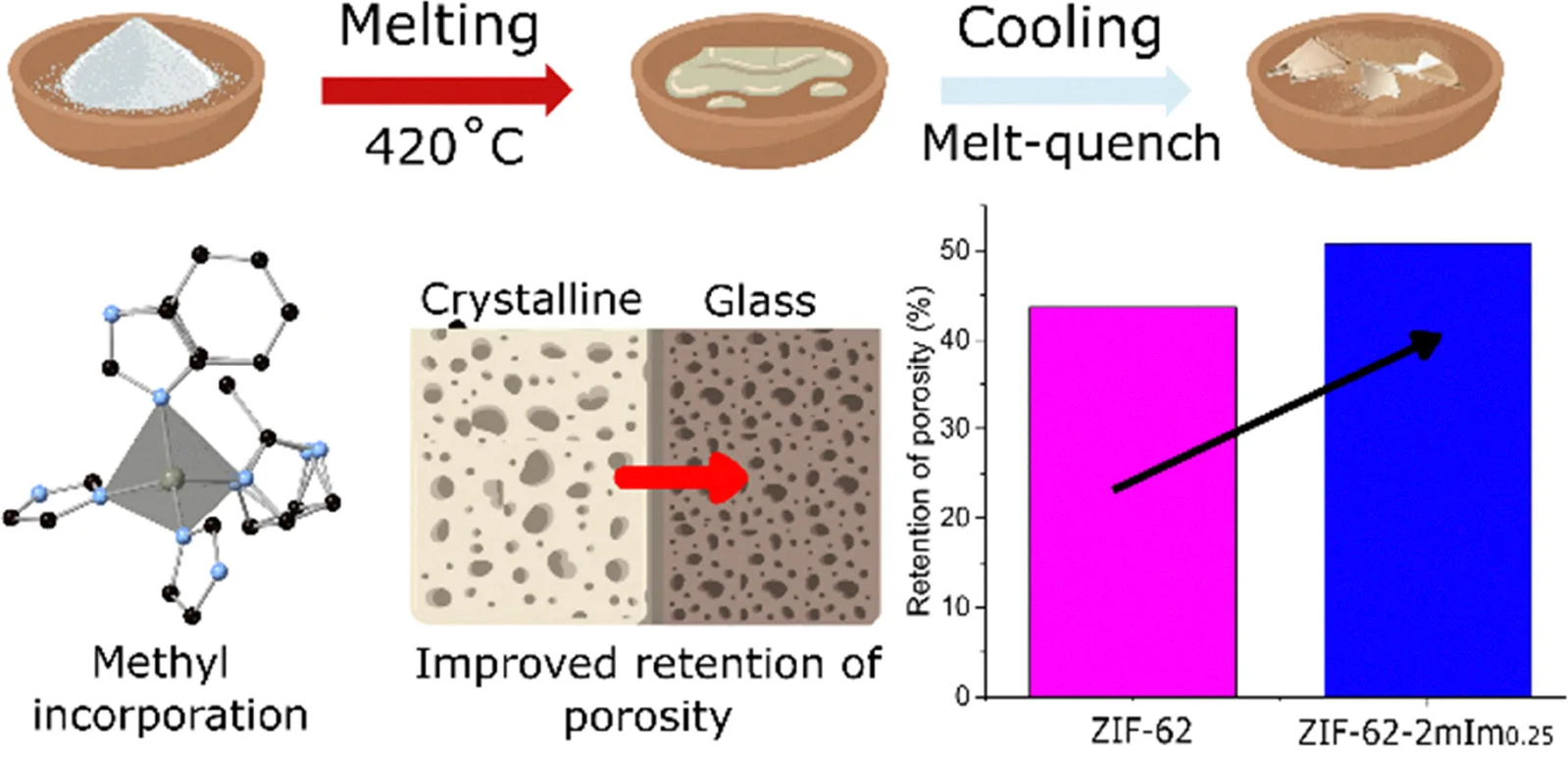

Zeolitic imidazolate frameworks (ZIFs), a subfamily of metal–organic frameworks (MOFs), are materials with zeolite-type topology, consisting of tetrahedrally coordinated metal ions with imidazole-based linkers.^1^ These materials have gained widespread attention due to their favourable adsorption and separation properties.^2^ An interesting quality of certain ZIF materials is their glass-forming ability (GFA), the ability to avoid crystallisation upon cooling from the melt, forming an amorphous glass (*a*_g_) ZIF.^3^ This GFA offers a route to overcoming the poor mechanical stability and limited processability associated with crystalline porous materials.^4^

Among glass-forming ZIFs, ZIF-62 [Zn(Im)_1.75_(bIm)_0.25_] (Im = imidazole, bIm = benzimidazole) has been extensively explored due to its exceptional GFA.^5^ However, despite the promise of ZIF-derived glasses, vitrification is accompanied by a substantial reduction in accessible porosity relative to the parent crystalline framework as a result of bonds reordering.^6^ Consequently, strategies to enhance the porosity in ZIF glasses are necessary for their widespread application. Linker modification offers a potential solution; where incorporating bulkier ligands may increase free volume and accessible porosity through steric expansion of the framework.^7^ Small additions could enhance porosity while retaining GFA, whereas excessive incorporation may hinder melting or favour decomposition over vitrification; therefore, a balance is necessary.

Mixed-linker ZIFs provide an attractive platform for exploring the relationship between linker sterics, framework disorder, and vitrification. The influence of linker variation on thermal behaviour is demonstrated by the difference between ZIF-4 [Zn(Im)_2_] and ZIF-62. Where ZIF-4 consists of only Im linkers, the presence of bIm linkers in ZIF-62 results in a lower melting temperature and improved GFA.^8^ Similar effects have been explored in ZIFs containing substituted benzimidazole derivatives such as chlorobenzimidazole or methylbenzimidazole, where increased linker size and asymmetry influence melting and framework packing.^9^

Building on this concept, this work investigates the incorporation of a third linker into ZIF-62 to perturb framework packing and potentially increase porosity while retaining GFA. 2-Methylimidazole (2-mIm), with a van der Waals volume (*V*_vdW_) larger than Im and smaller than bIm (Fig. 1a.), was selected for substitution with Im. Previous studies have explored substitution of bIm;^7^ however, we hypothesise that replacing the Im ligand will result in a ZIF with a lower melting temperature and enhanced processability. As substitution of Im with bIm has previously been shown to reduce the melting temperature of ZIFs, we anticipate a comparable effect upon substitution with 2-mIm, allowing for additional free volume, without significantly disrupting the framework and hindering GFA. Previous mixed-linker work with solvent-assisted linker exchange has been employed on non-melting ZIF-8, resulting in promising three-linker glass-forming *a*_g_ZIF-8 derivatives.^10^ In this approach, we aim to mechanochemically introduce a third linker, exercising the stoichiometric control of this method. Mechanochemistry offers several advantages for mixed-linker ZIF synthesis, including efficient reagent incorporation, reduced solvent use, and access to metastable or compositionally complex frameworks that may be difficult to obtain through conventional solvothermal methods.^11^ Solvothermal synthesis was employed to obtain single crystals (SC) to be used solely for structural investigations (see methods section in the SI).

**Fig. 1:**
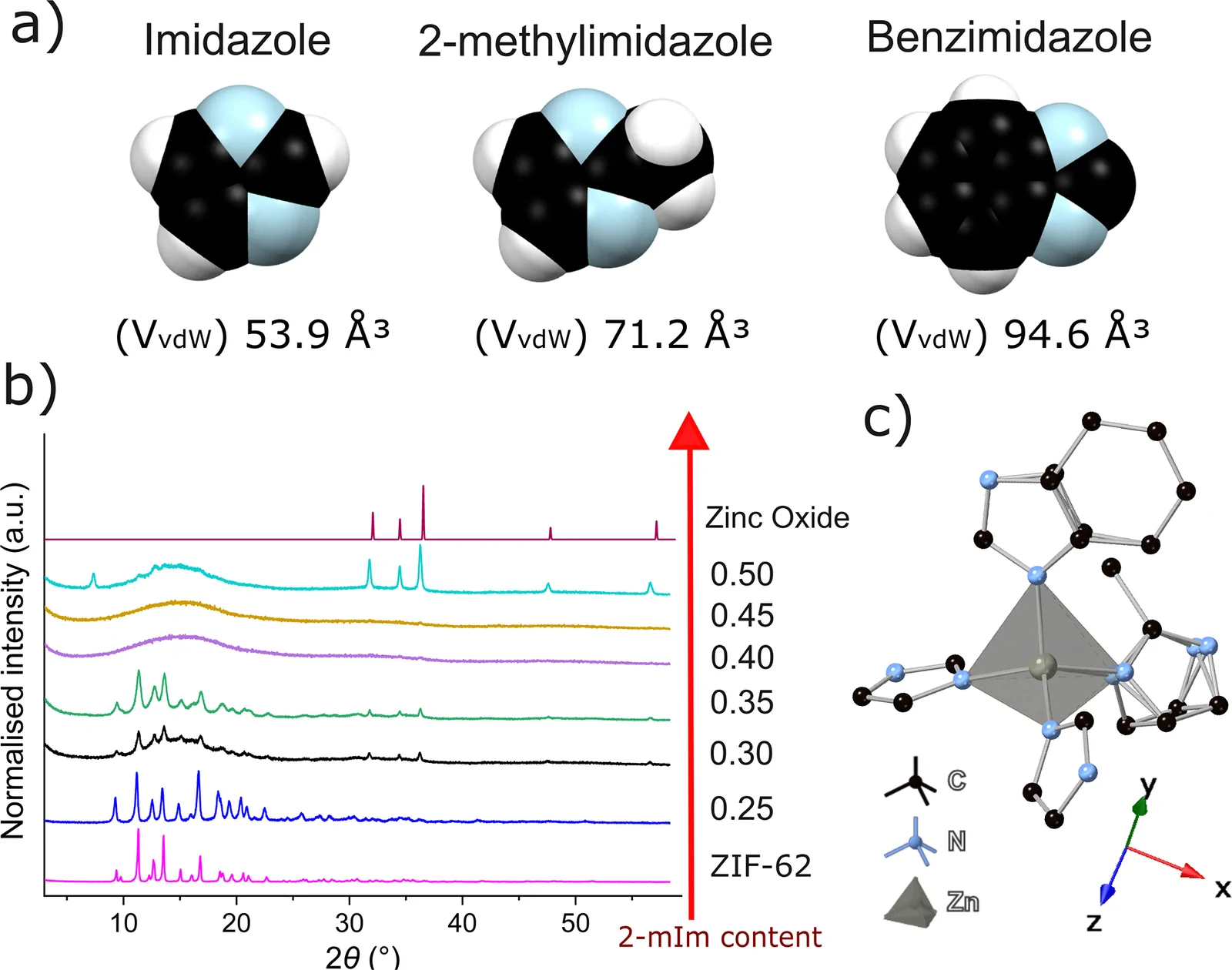
(a) Schematic of imidazole, 2-methylimidazole, and benzimidazole, and their van der Waals volumes (*V*_vdW_), calculated with a formula developed by Zhao *et al.*^12^ (b) Stacked PXRD patterns of ZIF-62, compositions [Zn(Im)_1.75−*x*_(2-mIm)_*x*_(bIm)_0.25_] (*x* = 0.25, 0.3, 0.35, 0.4, 0.45 and 0.5), and zinc oxide pattern. (c) Structural unit of the solvothermally synthesised three-linker ZIF [Zn(Im)_1.30_(2-mIm)_0.05_(bIm)_0.65_].

Initially, compositions [Zn(Im)_1.75−*x*_(2-mIm)_*x*_(bIm)_0.25_] with substitutions of *x* = 0.25 to 0.5 were synthesised mechanochemically. Above a 2-mIm substitution of 0.25, the synthesised materials displayed clear broad peaks and large zinc oxide (ZnO) peaks in their powder X-ray diffraction (PXRD) patterns, indicating unreacted ZnO present (Fig. 1b) and amorphisation before formation. This suggests that only limited incorporation can be accommodated without compromising structural integrity, likely due to the increased steric hinderance of the methyl group. [Zn(Im)_1.50_(2-mIm)_0.25_(bIm)_0.25_], subsequently referred to as ZIF-62-2mIm_0.25_, was found to be the maximum 2-mIm loading incorporated without hindered or incomplete formation. PXRD patterns in Fig. 1b, and Pawley fits (Fig. S1 and S2) illustrate the high crystallinity and ZIF-like structure of ZIF-62-2mIm_0.25_. While PXRD allows for differentiation between crystalline and glass forms (Fig. S3), potential changes in connectivity or structure disruption may not be detectable by PXRD alone. To evidence three linker incorporation, the single crystals were analysed using single crystal X-ray diffraction (SCXRD), which showed that 2-mIm was incorporated into the structure (Fig. 1c) (elaborated in the SI Fig. S4–S6).

While PXRD and SCXRD confirmed crystallinity and phase composition, these methods cannot determine the concentration of 2-mIm, with occupancies calculated from SCXRD affected by factors such as disordered scattering. Therefore, solution-state ^1^H NMR (elaborated in the SI Fig. S7–14)^13^ and ^13^C magic angle spinning (MAS) NMR spectroscopy were employed to enable a more direct evaluation of the extent the introduced linker is present.

Quantitative directly excited ^13^C MAS NMR spectra (Fig. 2, Fig. S15) and ^13^C cross-polarisation (CP) MAS NMR spectra (Fig. S16–S18) of ZIF-62-2mIm_0.25_ and SC-ZIF-62-2mIm showed incorporation of 2-mIm as revealed by the observation of signals at 13.2 ppm and 12.9 ppm, respectively, in good agreement with those reported for the CH_3_ group of 2-mIm in ZIF-8.^14,15^ Additionally, a small shoulder was observed, likely as a result of occupancies within distinct tetrahedra as has been previously reported for ZIF-62 (Further details given in SI).^5^ Spectral deconvolution of the quantitative directly excited ^13^C MAS NMR spectrum (Fig. S15) enabled the ligand stoichiometry of ZIF-62-2mIm_0.25_ and SC-ZIF-62-2mIm_0.05_ to be determined (Full description in the SI). The ligand stoichiometry of ZIF-62-2mIm_0.25_ was determined to be (Im)_1.60_(2-mIm)_0.21_(bIm)_0.19_, showing an approximately 1 : 1 ratio between 2-mIm and bIm in agreement with the nominal composition of (Im)_1.50_(2-mIm)_0.25_(bIm)_0.25_. In sharp contrast, SC-ZIF-62-2mIm_0.05_ contained a linker ratio of (Im)_1.30_(2-mIm)_0.05_(bIm)_0.65_, aligning with the values observed with ^1^H NMR (elaborated in the SI), furthermore, defining this sample as SC-ZIF-62-2mIm_0.05_. This large deviation in stoichiometry arises due to the solvothermal preference for bIm.^16^ This is avoided with mechanochemical methods, which provide more precise stoichiometric control.

**Fig. 2:**
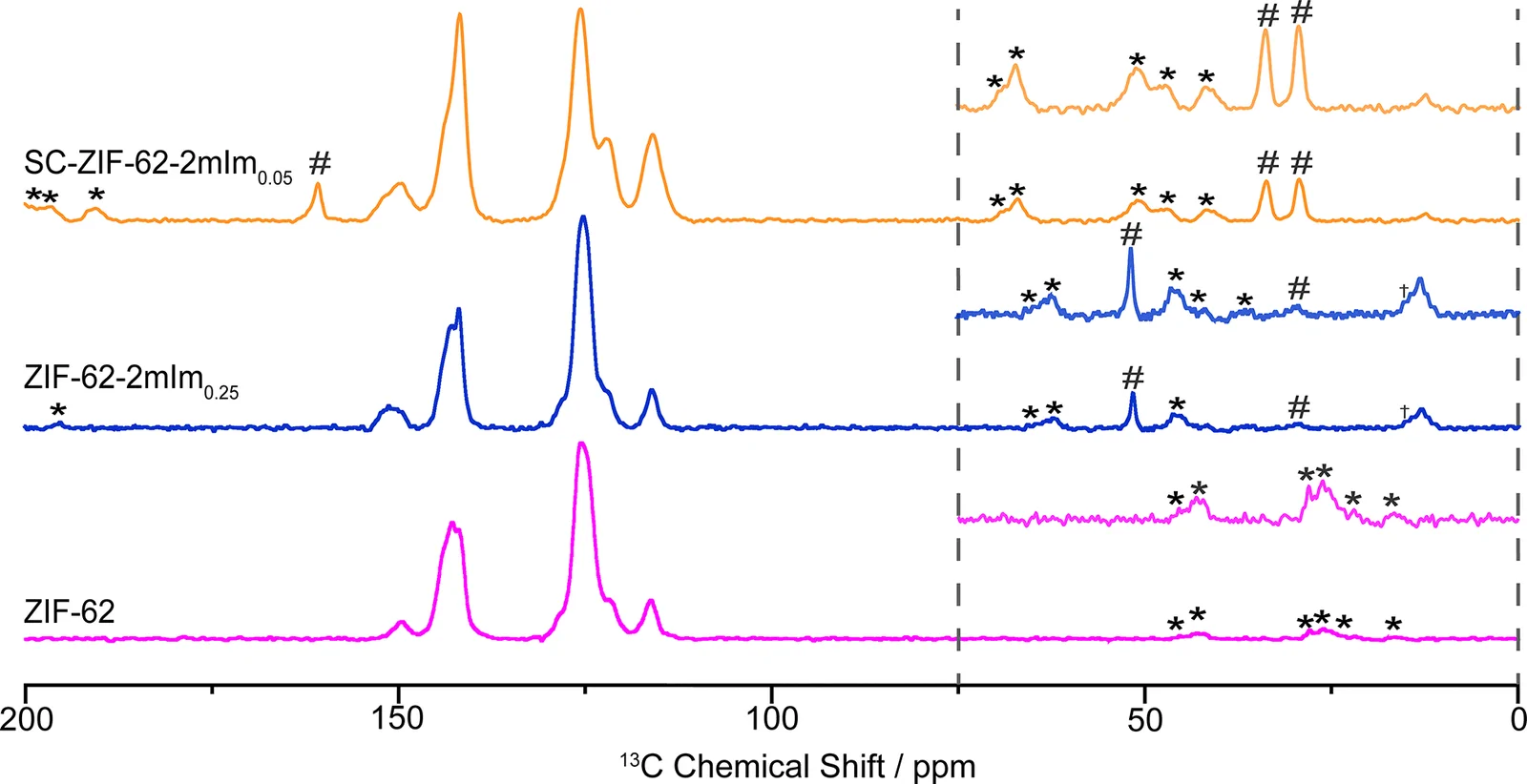
Directly excited ^13^C MAS NMR spectra of SC-ZIF-62-2mIm_0.05_, ZIF-62-2mIm_0.25_, and ZIF-62 obtained at 9.4 T, with magnified views of the spectra in the 75 to 0 ppm region. Spinning sidebands, identified from ^13^C CP spectra at variable MAS (Fig. S18) are marked with asterisks (*), a dagger (†) symbol highlights the potential second coordination site of 2-mIm (Deconvolution details are elaborated in the SI, Fig. S15), and hashtags (#) show residual solvent DMF and dichloromethane at 29–33 and 161 ppm and 51.9 ppm, respectively (further details in Table S8).

This deviation may explain the slight change in chemical shift for the CH_3_ group in 2-mIm between ZIF-62-2mIm_0.25_ and SC-ZIF-62-2mIm_0.05_, as the local environment is substantially different. ZIF-62 derivatives with high bIm compositions have been previously reported, and the steric hinderance caused by the excess bIm linker may explain the low incorporation of the bulky 2-mIm linker within SC-ZIF-62-2mIm_0.05_.^5,17^ Overall, these results strongly support the 2-mIm linker coordination to Zn^2+^, forming a three-linker ZIF.

To evaluate the thermal stability of the newly synthesised three-linker ZIF, thermogravimetric analysis (TGA) was performed (Fig. 3a). Overall, the samples displayed comparable degradation profiles and residual mass at 1273 K. ZIF-62-2mIm_0.25_ exhibited an onset decomposition temperature (*T*_d_) of around 848 K, which was slightly lower than that observed for ZIF-62, at around 868 K. The decomposition process also occurred more gradually, which may indicate differing framework interactions. However, the presence of a bulkier methyl substituent may introduce structure destabilising steric effects, thus, lowering *T*_d_. The results indicate that incorporation of 2-mIm does not significantly affect the bulk thermal stability of the framework. This is consistent with previous studies of mixed-linker ZIF systems, in which linker substitution typically exerts minimal influence on overall thermal robustness.^18^ Following the determination of the *T*_d_, differential scanning calorimetry (DSC) was conducted to identify key thermal transitions (Fig. S19–S20). ZIF-62-2mIm_0.25_ displayed an endothermic peak corresponding to the melt, and a melting temperature (*T*_m_) of 623 K during the first heating cycle, whereas ZIF-62 displayed a *T*_m_ at around 653 K (Fig. 3b), aligning with the thermal behaviour observed by TGA, likely due to destabilisation from the methyl group. In the second heating cycle shown (Fig. 3c), a step change in the baseline with an onset at 599 K indicated the glass transition temperature (*T*_g_) of ZIF-62-2mIm_0.25_, which is higher than that of ZIF-62 (588 K).

**Fig. 3:**
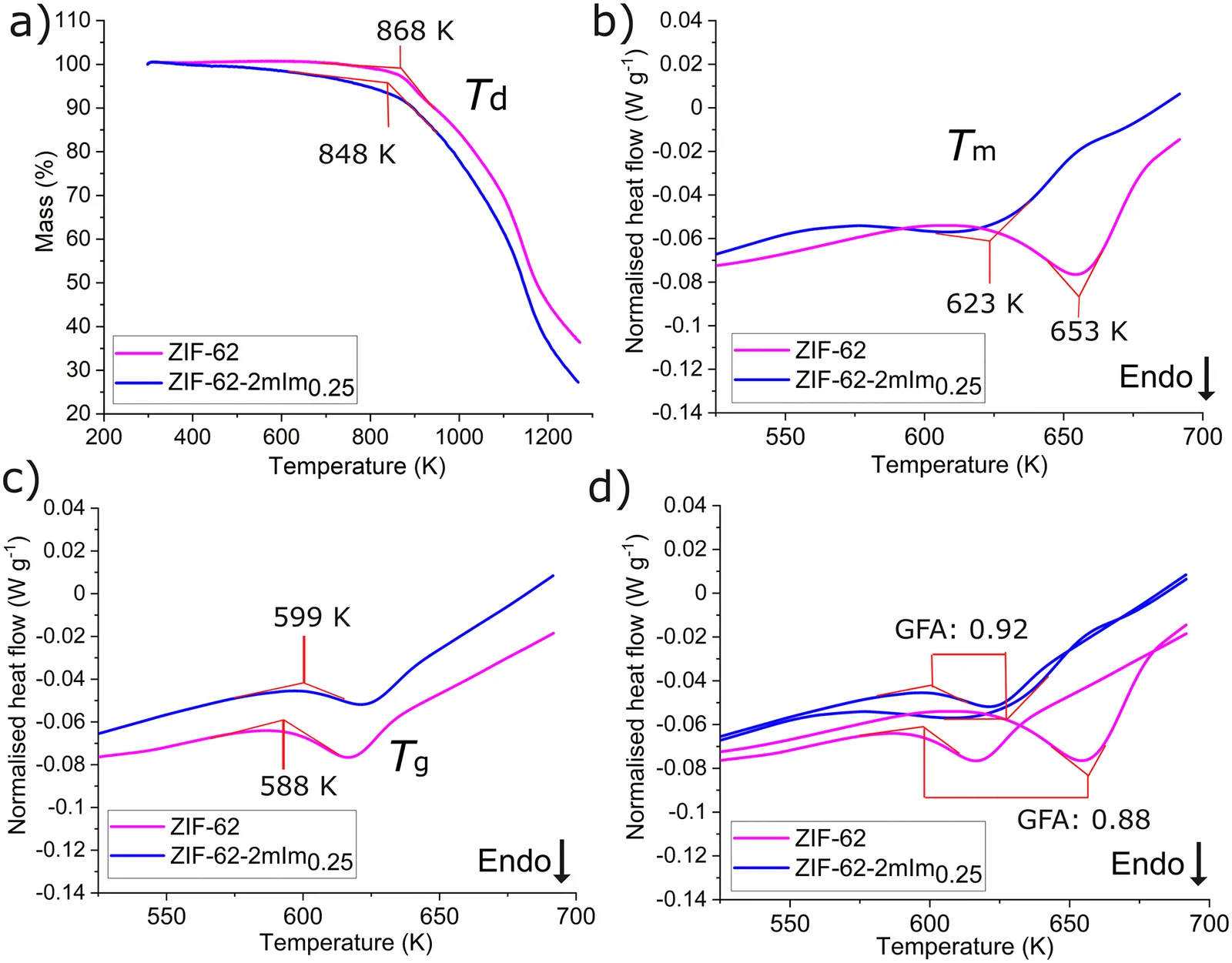
(a) TGA decomposition profiles with the onset *T*_d_ values. Magnified views of (b) the melting peaks and (c) the glass transitions. (d) Overlay of the *T*_m_ and *T*_g_ to illustrate the GFA of ZIF-62 (magenta) and ZIF-62-2mIm_0.25_ (blue), respectively.

Ultimately, ZIF-62-2mIm_0.25_ displayed a lower *T*_m_ and higher *T*_g_, allowing for a facile melt quenching process. A factor in processability is the GFA of a material, which can be measured by the ratio *T*_g_/*T*_m_ in Kelvin. It was found to be 0.92 for ZIF-62-2mIm_0.25_ and 0.88 for ZIF-62 (Fig. 3d). A GFA above 2/3 is considered high, thus, a GFA of 0.92 is a notable value, further evidencing feasible processability.^5^ Additionally, with a lower *T*_m_ and *T*_d_, the risk of decomposition upon melting is significantly reduced. These results confirm that the incorporation of 2-mIm does not inhibit glass formation, as the characteristic *T*_m_ and *T*_g_ remain clearly defined despite substitution with a bulkier linker, yielding a new glass-forming three-linker ZIF composition.

Following confirmation of glass formation, samples of *a*_g_ZIF-62-2mIm_0.25_ were synthesised *via* melt-quenching using a tube furnace heated to 693 K under an argon atmosphere. PXRD analysis of *a*_g_ZIF-62-2mIm_0.25_ displayed a broad diffuse hump between 10–30 2*θ* (°), characteristic of amorphous materials (Fig. S3). To investigate the structural differences from incorporating an additional linker, pair distribution function (PDF) data were collected (Fig. 4 and Fig. S21–28). Both ZIF-62 and ZIF-62-2mIm_0.25_ were analysed and displayed broadly similar PDF patterns.^19^ However, ZIF-62-2mIm_0.25_ displayed a different ratio of peak sizes, likely due to the presence of 2-mIm. Crystallographic information files (CIFs) of ZIF-62^20^ and SC-ZIF-62-2mIm_0.05_ were used to assign individual atom-atom correlations through weighted partial PDF analysis (Fig. S22 and S26), with consideration of the lower 2-mIm content of the single crystal sample.^21^ The presence of the methyl group in ZIF-62-2mIm_0.25_ resulted in an increased contribution from C–C bonds (Fig. 4a), giving rise to a more intense first peak. This observation is consistent with previous reports showing a large first peak (∼1.3 Å) and smaller second (∼2 Å) observed for 2-mIm.^22^ A significant peak at 5 Å was observed for ZIF-62-2mIm_0.25_, compared with a smaller peak for ZIF-62. This may be attributed to the differing compositions of the mechanochemical samples. Comparisons of the crystalline and glassy phases revealed that, for *a*_g_ZIF-62, the short-range correlations up to ∼6 Å, remained nearly unchanged indicating a preservation of the local structure upon vitrification (Fig. 4b).

**Fig. 4:**
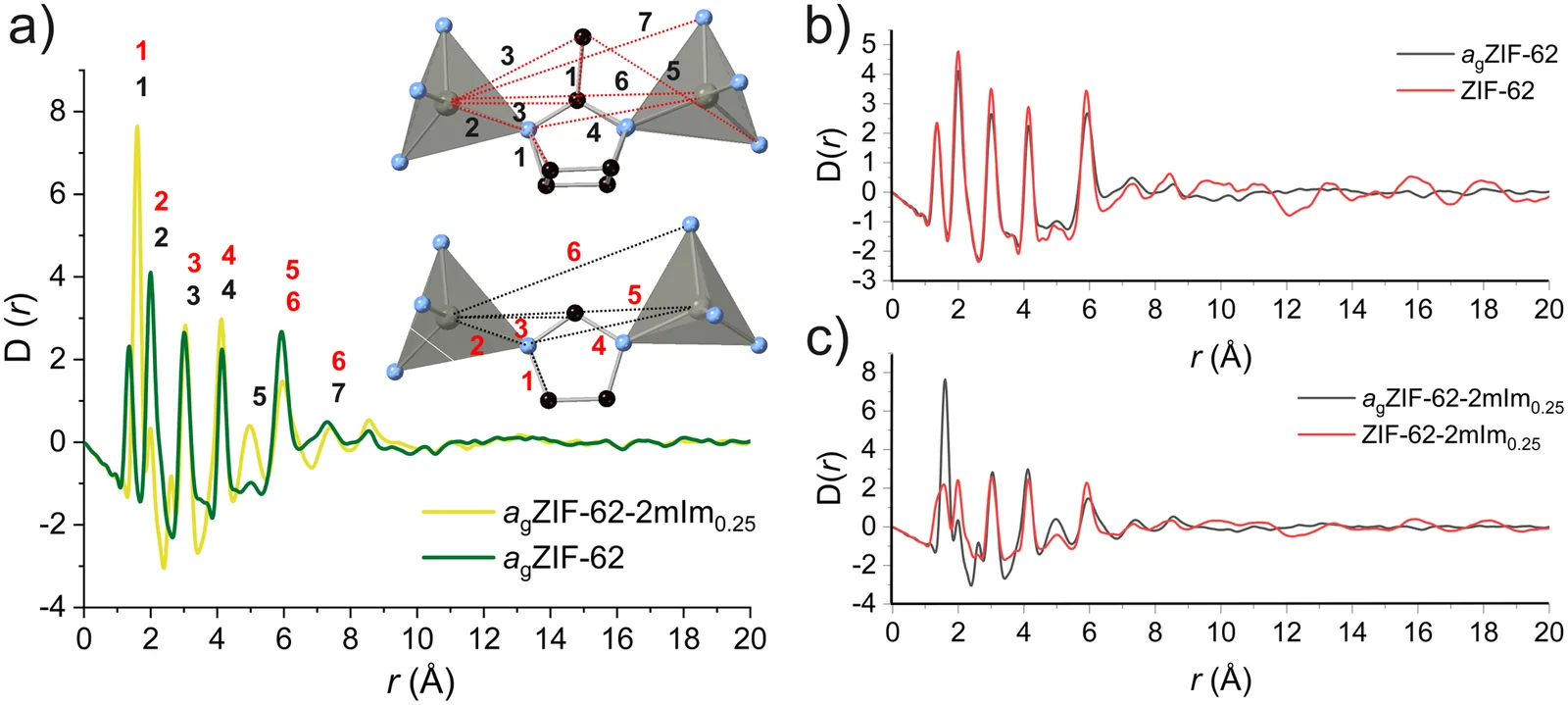
(a) *D*(*r*) of *a*_g_ZIF-62 and *a*_g_ZIF-62-2mIm_0.25_ showing the correlations of the Zn-Im-Zn and Zn-2-mIm-Zn bonding units in green and yellow, respectively (b). *D*(*r*) of *a*_g_ZIF-62 and ZIF-62 in black and red, respectively (SI Fig. S25 and S27 (c) *D*(*r*) of *a*_g_ZIF-62-2mIm_0.25_ and ZIF-62-2mIm_0.25_ in black and red, respectively (SI Fig. S21 and S23).

In contrast, although the peaks for ZIF-62-2mIm_0.25_ and *a*_g_ZIF-62-2mIm_0.25_ mostly overlap, these variations suggest structural differences between phases, likely associated with the additional third linker and the subsequent influence on the local atomic environment (Fig. 4c).

While nitrogen (N_2_) gas sorption is commonly used, ZIF-62 has consistently been shown to not adsorb N_2_, thus, these measurements were not performed.^23^ Previous work has shown that carbon dioxide (CO_2_) measurements performed at 195 K provide deeper insights into the porosity of ZIF glasses, allowing for determination of the pore sizes.^24^ Therefore, we performed CO_2_ isotherms on ZIF-62, ZIF-62-2mIm_0.25_, and their corresponding glasses to determine their respective uptakes (Table S13). The crystalline ZIF-62-2mIm_0.25_ CO_2_ isotherm (Fig. 5a) displayed an uptake of 117.7 cm^3^ g^−1^. This marks a 7.8% reduction in the CO_2_ uptake relative to ZIF-62 (127.7 cm^3^ g^−1^), thus evidencing that with the incorporation of a bulkier ligand, the CO_2_ uptake is hindered by the 2-mIm blocking accessible pore space. However, when comparing the CO_2_ uptake at 273 K, the values are higher than previously reported methyl benzimidazole-containing three-linker glass-forming ZIFs in their crystalline states, with values and figures highlighted in the SI (Table S14 and Fig. S29–S30).^7^ The *a*_g_ZIF-62-2mIm_0.25_ CO_2_ isotherm showed a higher uptake than *a*_g_ZIF-62 (59.3 cm^3^ g^−1^), with a value of 66.1 cm^3^ g^−1^ marking an 11.4% increase. While *a*_g_ZIF-62-2mIm_0.25_ shows an increase over *a*_g_ZIF-62, other three-linker glass-forming systems, derived from a solvent assisted linker exchange of ZIF-8, have shown higher uptakes, highlighting the further development required in mechanochemically synthesised three-linker glass-forming ZIFs.^10^ Where ZIF-62 displays a 50.8% reduction upon glass formation, ZIF-62-2mIm_0.25_ has a reduction of 43.6%, therefore illustrating the enhanced retention of porosity.

**Fig. 5:**
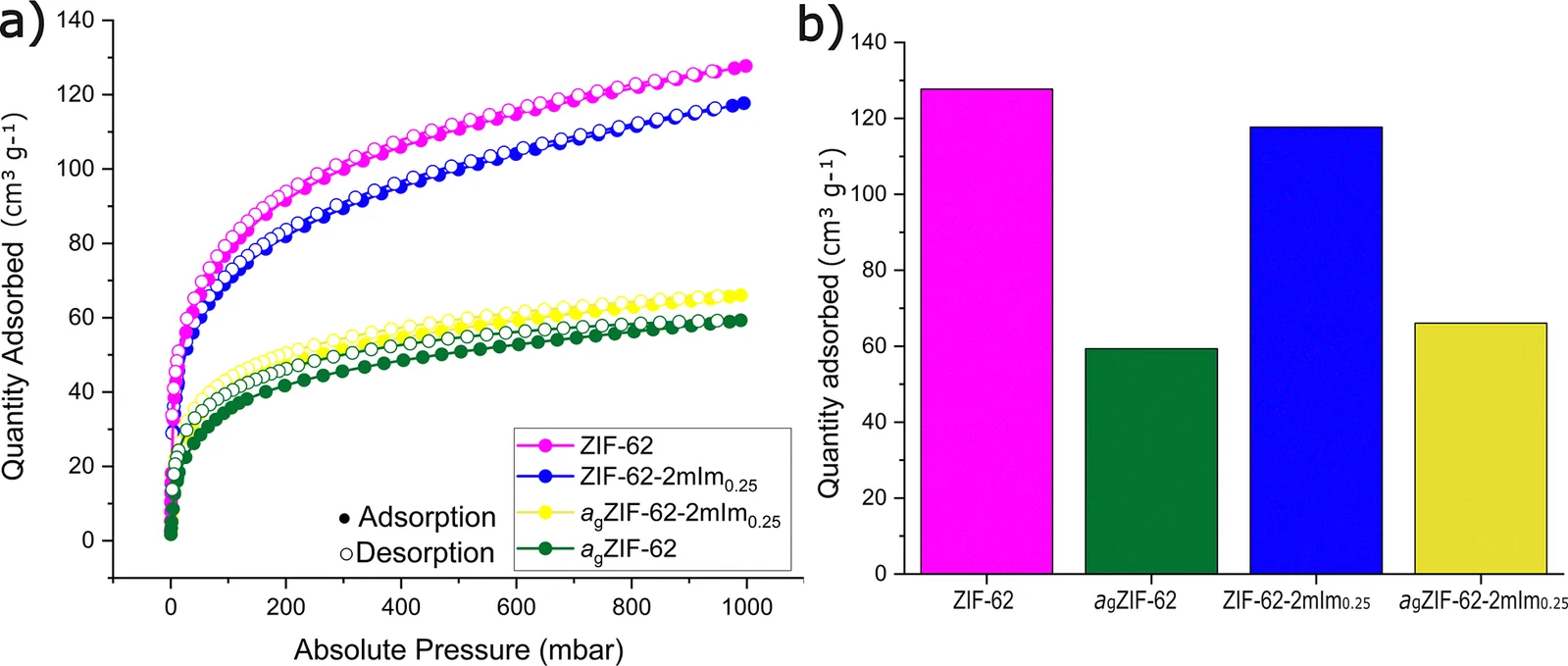
(a) CO_2_ adsorption-desorption isotherms performed at 195 K for ZIF-62, ZIF-62-2mIm_0.25_, a_g_ZIF-62, and a_g_ZIF-62-2mIm_0.25_. (b) a bar chart illustrating the maximum uptake values for ZIF-62, a_g_ZIF-62, ZIF-62-2mIm_0.25_, and a_g_ZIF-62-2mIm_0.25_.

In this work, we mechanochemically synthesised a three-linker glass forming ZIF (ZIF-62-2mIm_0.25_), broadening the landscape of amorphous MOFs. NMR experiments provided confirmation of the 2-mIm incorporation, which was supported by crystallographic evidence. We show that by substituting Im for 2-mIm, we promote structural destabilisation as highlighted by the lower *T*_m_ and earlier *T*_d_ observed in the thermal analysis for ZIF-62-2mIm_0.25_. However, despite the added steric hinderance, the GFA for ZIF-62-2mIm_0.25_ is preserved. PDF measurements illustrated the bonding and interactions within ZIF-62-2mIm_0.25_, highlighting the interactions unique to a three-linker glass-forming ZIF. Through CO_2_ uptake measurements we demonstrate that linker selection directly influences porosity in both crystalline and glassy states. Substitution with 2-mIm reduces CO_2_ uptake in the crystalline form, consistent with steric hindrance limiting pore accessibility. In contrast, upon glass formation there is an increased uptake, highlighting an improved retention of porosity for ZIF-62-2mIm_0.25_ over ZIF-62. This suggests that the additional methyl group blocks uptake in the crystalline phase but preserves porosity in the glass. Overall, this work establishes the influence of a three-linker composition on the thermal properties and CO_2_ uptake of a three-linker glass-forming ZIF, providing foundational insights for the design and development of multi-linker MOF glasses.

## Author contributions

LNM; conceptualisation, funding acquisition, project administration, supervision, writing – original draft, review and editing. BT; conceptualisation, investigation, PXRD, thermal, NMR, and sorption analysis and processing, PDF processing, writing – original draft, review and editing. HGP; solid-state NMR analysis, writing – review and editing. SDH; SCXRD collection and analysis, writing – review and editing. JM; SCXRD collection, investigation, writing – review and editing. CY; PDF collection and processing. AL; PDF collection. LL; XRD collection. FB; funding acquisition, supervision, writing – original draft, review and editing.

## Conflicts of interest

There are no conflicts to declare.

## Supplementary Material

CC-OLF-D6CC04372K-s001

## Data Availability

The data supporting this article have been included as part of the supplementary information (SI). Supplementary information includes synthesis details, methods, ^1^H NMR, SS-NMR, Thermal data (TGA, DSC), Crystallographic data (PXRD, SCXRD, PDF) and CO_2_ gas sorption data. See DOI: https://doi.org/10.1039/d6cc04372k. Data for this article, including NMR, MAS NMR, DSC, PXRD, PDF, TGA, SCXRD and gas sorption isotherms are available at The University of Liverpool's Data Catalogue at DOI: https://doi.org/10.17638/datacat.liverpool.ac.uk/3094. CCDC 2566200 contains the supplementary crystallographic data for this paper.^25^
