# Supplementary material for: Enhanced retention of porosity in a three-linker glass-forming zeolitic imidazolate framework
Source: Chem Commun (Camb). 2026 Oct 29. Online ahead of print. doi: 10.1039/d6cc04372k (PMC13749207; doi:10.1039/d6cc04372k)
Supplement: Supplementary file 1 [file CC-OLF-D6CC04372K-s001.pdf]

Supplementary Information (SI) for Chemical Communications.
This journal is © The Royal Society of Chemistry

Supplementary Information

Enhanced Retention of Porosity in a Three-Linker Glass-Forming Zeolitic Imidazolate Framework

Bethan Turner,[a] Harry G. Palmer,[a] Sam D. Harding,[b] Jay McCarron,[a] Chumei Ye,[c] Arad Lang,[d, e] Louis Longley,[b] Frédéric Blanc,[a,f,g] and Lauren N. McHugh*[a]

*Email: L.N.Mchugh@liverpool.ac.uk

[a] Department of Chemistry, University of Liverpool, Liverpool L69 7ZD, U.K.

[b] Materials Innovation Factory, University of Liverpool, Liverpool L69 7ZD, U.K

[c] Department of Chemistry, Graduate School of Science, Kyoto University, Kitashirakawa-Oiwakecho, Sakyo-ku, Kyoto 606-8502, Japan.

[d] Department of Materials Science and Metallurgy, University of Cambridge, Cambridge CB3 0FS, U.K.

[e] Department of Materials Science and Engineering, Technion – Israel Institute of Technology, Haifa 3200003, Israel.

[f] Leverhulme Research Centre for Functional Materials Design, UK;

[g] Stephenson Institute for Renewable Energy, University of Liverpool, Liverpool L69 7ZF, U.K.

Table of Contents

1. Synthetic methods **3**

1.1 Materials 3

1.2 Mechanochemical synthesis of ZIF-62 3

1.3 Mechanochemical synthesis of ZIF-62-2mIm$_{0.25}$ 3

1.4 Solvothermal synthesis of ZIF-62 3

1.5 Solvothermal synthesis of SC-ZIF-62-2mIm$_{0.05}$ 3

2. Experimental methods **5**

2.1 ^{1}H Solution nuclear magnetic resonance (NMR) 5

2.2 Solid-state nuclear magnetic resonance (NMR) 5

2.3 Thermogravimetric Analysis (TGA) 5

2.4 Differential scanning calorimetry (DSC) 6

2.5 Single Crystal X-ray Diffraction (SCXRD) 6

2.6 Powder X-ray diffraction (PXRD) 6

2.7 X-ray Total Scattering and Pair Distribution Function (PDF) Analysis 7

2.8 PDFgui 7

2.9 Gas Sorption Analysis 7

3. Material characterisation and analysis **8**

3.1 Powder X-ray diffraction (PXRD) 8

3.2 Single crystal X-ray diffraction (SCXRD) 11

3.3 Nuclear Magnetic Resonance (NMR) spectroscopy 14

3.3.1 ^{1}H NMR 14

3.3.2 Solid -state NMR 22

3.4 Differential scanning calorimetry (DSC) 27

3.5 Pair distribution function (PDF) 28

3.6 CO_2 Gas sorption 34

4. References 36

1. Synthetic Methods

1.1 Materials

Imidazole (≥ 99.5 %), Benzimidazole (98 %), Zinc oxide (ZnO), zinc acetate dihydrate ($Zn(OAc)_2.2H_2O$), DMSO-d_6, deuterium chloride solution (DCl, 35 wt. % in D_2O, ≥ 99 atom% D), zinc nitrate hexahydrate ($Zn(NO_3)_2·6H_2O$) (98 %), and 2-methyl imidazole were purchased from Sigma-Aldrich. N,N-Dimethylformamide (DMF), dichloromethane (DCM), and acetone were purchased from Fisher Scientific.

1.2 Mechanochemical synthesis of ZIF-62

Imidazole (596 mg, 8.75 mmol), benzimidazole (148 mg, 1.25 mmol), ZnO (<100 nm) (402.96 mg, 4.95 mmol), and $Zn(OAc)_2·2H_2O$ (9.17 mg, 0.05 mmol) were weighed and transferred into a 50 mL stainless steel ball mill jar. DMF (500 µL) was added, followed by the addition of two 20 mm stainless steel grinding balls. The sealed jar was shaken at 30 Hz for 30 minutes using a Retsch MM400 Mixer Mill. Post-synthesis, the resulting powders were recovered and washed by vacuum filtration using DMF (3 × 25 mL) followed by DCM (25 mL). To complete solvent exchange, the powders were soaked in DCM (50 mL) for 24 hours. Samples were then activated under dynamic vacuum at 170 °C for 3 hours to remove residual solvent from the pores and cooled to room temperature under vacuum prior to characterisation.

1.3 Mechanochemical synthesis of ZIF-62-2mIm$_{0.25}$

Imidazole, 2-methylimidazole, benzimidazole, ZnO, and $Zn(OAc)_2·2H_2O$ were weighed (with precise reagent quantities outlined in Table S1) and transferred into a 50 mL stainless steel ball mill jar. DMF (500 µL) was added, followed by the addition of two 20 mm stainless steel grinding balls. The sealed jar was shaken at 30 Hz for 30 minutes using a Retsch MM400 mixer mill. Post-synthesis, the resulting powders were recovered and washed by vacuum filtration using DMF (3 × 25 mL) followed by DCM (25 mL). To complete solvent exchange, the powders were soaked in DCM (50 mL) for 24 hours. Samples were then activated under dynamic vacuum at 170 °C for 3 hours to remove residual solvent from the pores and cooled to room temperature under vacuum prior to characterisation.

1.4 Solvothermal synthesis of ZIF-62

$Zn(NO_3)_2·6H_2O$ (4.0 mmol, 1.2 g), imidazole (11.55 mmol, 786 mg) and benzimidazole (1.660 mmol, 196 mg) were weighed, then dissolved in DMF to yield a total volume of 15 mL, ultrasonicated for 5 minutes, and transferred to a 100 mL borosilicate screw top jar. The tightly sealed reaction vials were heated for 6 days at 110 °C. After cooling, the reaction mixtures were filtered and washed DMF (3 x 20 mL). Subsequently, the crystals were solvent exchanged in DCM (40 mL) for 2 days. After filtration, the single crystals (SC) were dried at 170 °C under a dynamic vacuum overnight.

1.5 Solvothermal synthesis of SC-ZIF-62-2mIm$_{0.05}$

$Zn(NO_3)_2·6H_2O$, imidazole, 2-methylimidazole, and benzimidazole (with precise quantities outlined in Table S2) were weighed, then dissolved in DMF to yield a total volume of 15 mL, ultrasonicated for 5 minutes, and transferred to a 100 mL borosilicate screw top jar. The tightly sealed reaction vials were heated for 6 days at 110 °C. After cooling, the reaction mixtures were filtered and washed with DMF (3 x 20 mL). Subsequently, the crystals were solvent exchanged in DCM (40 mL) for 2 days. After filtration, SC were dried at 170 °C under a dynamic vacuum overnight.

Table S1: Reagent quantities used for the synthesis of ZIFs with varying ratios of 2-mIm. Stoichiometries are based on the general formula $[Zn(Im)_{1.75}(bIm)_{0.25}]$ with a fixed amount of bIm and varying ratios of Im and 2-mIm.

Sample	ZnO	$Zn(OAc)_2.2H_2O$	Imidazole	Benzimidazole	2-Methylimidazole
**ZIF-62**	402.69 mg (4.95 mmol)	9.174 mg (0.05 mmol)	596 mg (8.75 mmol)	148 mg (1.25 mmol)	-
**$Zn(Im)_{1.50}(2\text{-}mIm)_{0.25}(bIm)_{0.25}$**	402.69 mg (4.95 mmol)	9.174 mg (0.05 mmol)	510.6 mg (7.5 mmol)	148 mg (1.25 mmol)	103 mg (1.25 mmol)
**$Zn(Im)_{1.45}(2\text{-}mIm)_{0.30}(bIm)_{0.25}$**	402.69 mg (4.95 mmol)	9.174 mg (0.05 mmol)	493.6 mg (7.25 mmol)	148 mg (1.25 mmol)	123.2 mg (1.5 mmol)
**$Zn(Im)_{1.40}(2\text{-}mIm)_{0.35}(bIm)_{0.25}$**	402.69 mg (4.95 mmol)	9.174 mg (0.05 mmol)	476.6 mg (7 mmol)	148 mg (1.25 mmol)	143.7 mg (1.75 mmol)
**$Zn(Im)_{1.35}(2\text{-}mIm)_{0.40}(bIm)_{0.25}$**	402.69 mg (4.95 mmol)	9.174 mg (0.05 mmol)	459.54 mg (6.75 mmol)	148 mg (1.25 mmol)	164.2 mg (2 mmol)
**$Zn(Im)_{1.30}(2\text{-}mIm)_{0.45}(bIm)_{0.25}$**	402.69 mg (4.95 mmol)	9.174 mg (0.05 mmol)	442.52 (6.5 mmol)	148 mg (1.25 mmol)	184.725 mg (2.25 mmol)
**$Zn(Im)_{1.25}(2\text{-}mIm)_{0.50}(bIm)_{0.25}$**	402.69 mg (4.95 mmol)	9.174 mg (0.05 mmol)	425.5 mg (6.25 mmol)	148 mg (1.25 mmol)	205.25 mg (2.5 mmol)

Table S2: Reagent quantities used for the solvothermal synthesis of ZIF-62 and 2-mIm mixed-linker analogues.

Sample	$Zn(NO_3)_2.6H_2O$	Imidazole	Benzimidazole	2-Methylimidazole	DMF	Temperature
**$Zn(Im)_{1.75}(BIm)_{0.25}$** (ZIF-62)	1.2 g (4 mmol)	0.786 g (11.55 mmol)	0.196 g (1.65 mmol)	-	15 mL	110 °C
**$Zn(Im)_{1.5}(2\text{-}mIm)_{0.25}(bIm)_{0.25}$**	1.2 g (4 mmol)	0.675 g (9.91 mmol)	0.196 g (1.65 mmol)	0.136 g (1.65 mmol)	15 mL	110 °C
**$Zn(Im)_{1.25}(2\text{-}mIm)_{0.50}(bIm)_{0.25}$**	1.2 g (4 mmol)	0.561 g (8,25 mmol)	0.196 g (1.65 mmol)	0.271 g (3.3 mmol)	15 mL	110 °C

2. Experimental Methods

2.1 ^{1}H Solution nuclear magnetic resonance (NMR)

Samples (*ca*. 10 mg) were digested in DCl(35%)/D_2O and D_2O and ^{1}H NMR spectra were recorded on a Bruker Advance 400 NMR spectrometer at 400.13 MHz. All spectra were analysed with MestReNova version 12. 0. 2-20910 software.

2.2 Solid state nuclear magnetic resonance (NMR)

All solid-state NMR measurements were recorded under Magic Angle Spinning (MAS) conditions on a Bruker Avance III HD NMR spectrometer with a 9.4 T external magnetic field, equipped with a 4 mm HXY triple-resonance MAS probe (used in double resonance mode) tuned to ^{1}H and ^{13}C Larmor frequencies of 400.15 MHz and 100.63 MHz on the X channel, respectively. The samples were finely ground and packed in air into 4 mm ZrO_2 rotors and closed with kel-f caps. All ^{1}H and ^{13}C were externally referenced to adamantane at chemical shifts of 1.8 ppm[1] and 29.45 ppm[2] (for the upfield -CH peak), respectively. All ^{13}C detection were performed with ^{1}H SPINAL-64 heteronuclear decoupling at a radio frequency (rf) field amplitude of 70 kHz.[3]

^{1}H - ^{13}C cross-polarisation (CP) MAS NMR spectra were obtained using initially ^{1}H excitation pulses at a rf field amplitude of 70 kHz.[3] The ^{1}H - ^{13}C CP step was achieved with a ^{13}C rf field amplitude of 48 kHz matched to linearly ramped (70-100%) ^{1}H at a rf field amplitude of approximately 50 kHz and spectra were recorded at multiple contact times. A recycle delay of at least 1.3 times the longest ^{1}H T_1 (determined by a saturation recovery experiment) was used for CP. Several spectra were obtained with multiple MAS frequencies to identify spinning sidebands (Fig. S18).

Directly excited ^{13}C MAS NMR spectra were collected for three ZIF samples with ^{13}C excitation pulses at a rf field amplitude of 50 kHz. MAS rates were chosen to allow for deconvolution of all sites without overlapping with sidebands (Table S8). To ensure quantitative data was obtained, directly excited ^{13}C MAS NMR experiments used recycling delays longer than five times the longest ^{13}C T_1 (determined using a CP inversion recovery experiment) which as in the order of 46-90 s (Table S8). ^{13}C T_1 times were obtained by integrating each region of the 1D spectrum to fit the signal amplitudes *I* to an equation of the form:

$$I = I_0\left(1 - 2\exp\left(-\frac{\tau}{T_1}\right)^{a}\right)$$

where I is the absolute signal intensity, I_0 is a scaling factor, τ the recovery delay, and a the exponential stretch factor.[4] The NMR data was processed using Topspin 5.0 and the data was fitted to this equation within the MATLAB 2025b Curve Fitter app. All directly excited ^{13}C MAS NMR spectra were deconvoluted using the solid line shape analysis tool “sola” in Topspin 5.0 to extract the relative linker ratios (Fig. S15).

2.3 Thermogravimetric Analysis (TGA)

TGA measurements were performed on a Netzsch TG 209F1 Libra. Samples (*ca*. 10 mg) in alumina crucibles were subjected to heating/cooling procedures outlined in Table S3 under a nitrogen (N_2) atmosphere.
All TGA data produced were analysed with Netzsch Proteus-Thermal Analysis 6.1.0 software.[5]

Table S3: TGA method

Initial Temperature / K	Final temperature / K	Rate / K min^{-1}	Atmosphere
**298**	1273	10	N_2

2.4 Differential scanning calorimetry (DSC)

DSC measurements were conducted on TA Instruments Discovery Differential Scanning Calorimeter. Samples (*ca*. 7 mg) were placed in Terzo aluminium hermetic pans and sealed with a pinhole lid. The samples were subjected to heating/cooling procedures shown in Table S4. All DSC data were analysed using TA Instruments TRIOS software version 5.1.1.

Table S4. DSC method

Step	Initial temperature / K	Final temperature / K	Rate /K min^{-1}
	**Equilibrate to 298 K**		
**Ramp**	303	693	10
**Ramp**	693	303	10
**Ramp**	303	693	10
**Ramp**	693	303	10
	**Isothermal hold for 1 minute**		

2.5 Single Crystal X-ray diffraction (SCXRD)

Single crystals of $Zn(Im)_{1.25}(2\text{-mIm})_{0.50}(bIm)_{0.05}$ were grown by method outlined in section 1.5 affording colourless, well defined polyhedra. A suitable crystal was harvested in a drop of mother liquor and mounted on a Mitegen loop. This was immediately transferred to the diffractometer and placed under an open flow helium cooling device (Oxford Cryosystems Helix) at 30 K. Intensity data were collected on beamline I19 (EH1) at Diamond Light Source (Didcot, UK) using silicon double crystal monochromated synchrotron radiation (λ = 0.6889 Å) and a Dectris Pilatus 2M detector. Data were collected using a combination of ω and ϕ scans, on a 3-circle (fixed κ) goniometer. Indexing, integration and absorption corrections were performed using the xia2 automated pipeline.[6] The structure was solved using SHELXT[7] and refined by full-matrix least-squares on $|F^2|$ using SHELXL,[8] integrated within Olex2.[9] All hydrogen atoms were placed geometrically and refined using a riding model. All heavy atoms were refined anisotropically. The SQUEEZE[10] routine of PLATON[11] was used to remove the scattering contribution of disordered solvent voids. Residual electron density after refinement was accounted for by further refining the data with rigid body DMF molecules in the pore. Uiso of the atoms in the DMF molecules were constrained to a common value through a free variable. For full refinement details, see Table S7; for images of the crystal structure see Fig. S4.

2.6 Powder X-ray difrraction (PXRD)

PXRD patterns ZIF-62, a_gZIF-62, ZIF-62-2mIm$_{0.25}$, and a_gZIF-62-mIm$_{0.25}$ were collected in vertical transmission mode from loose powder samples held on Mylar film in aluminium well plates, using a Panalytical Empyrean equipped with a high throughput screening XYZ stage, X-ray focusing mirror and PIXcel detector with Cu-Kα radiation (λ = 1.541 Å).

Simulated PXRD patterns were refined against experimental data using TOPAS academic (V.7) software.[12]

2.7 X-ray Total Scattering and Pair Distribution Function (PDF) Analysis

Total scattering experiments were performed at beamline I15-1 of Diamond Light Source, Oxfordshire, UK (experiment CY39316). A few mg of each sample were ground using a mortar and pestle and loaded into a Kapton capillary (inner diameter: 1 mm) which was then sealed. The samples were measured at room temperature, as the X-ray wavelength was set to be 0.161669 Å. An empty capillary and the blank instrument ("air") were also measured under the same conditions. Data was processed using the GudRunX software:[13] first, the data was corrected, accounting for absorption and scattering, and the background was reduced. Then, the resulting structure factor S(Q) was Fourier transformed (Q range: 21.1Å^{-1}), and the pair distribution function D(r) was produced.

2.8 PDFgui

PDF analysis was performed using PDFgui.[14] Collected CIF files of ZIF-62 and ZIF-62-2mIm$_{0.25}$ were utilised to identify the interatomic contributions within the structure. The Zn-Im-Zn and Zn-mIm-Zn units were studied and calculated individual atom-atom interactions were plotted and used as a reference for assignments.

2.9 Gas Sorption Analysis

The gas uptake measurements and adsorption analysis of CO_2 up to 1100 mbar at 273 K and up to 1000 mbar at 195 K were performed on a Micrometric 3-Flex 3500 Gas Sorption Analyser. Samples of ca. 100 mg were degassed in situ with N_2 by heating to 383 K at a rate of 10 K min^{-1} under vacuum of 10^{-4} mbar for 10 hours. During analysis, the temperature was controlled using a Micromeritics iso-controller chiller and cryostat.

Adsorption and desorption Isotherms were produced, providing values for the maximum uptake and trends in adsorption/desorption. Analysis was performed with Micromeritics Flex Version 6.03 software.

3. Material characterisation and analysis

3.1 Powder X-ray Diffraction (PXRD)

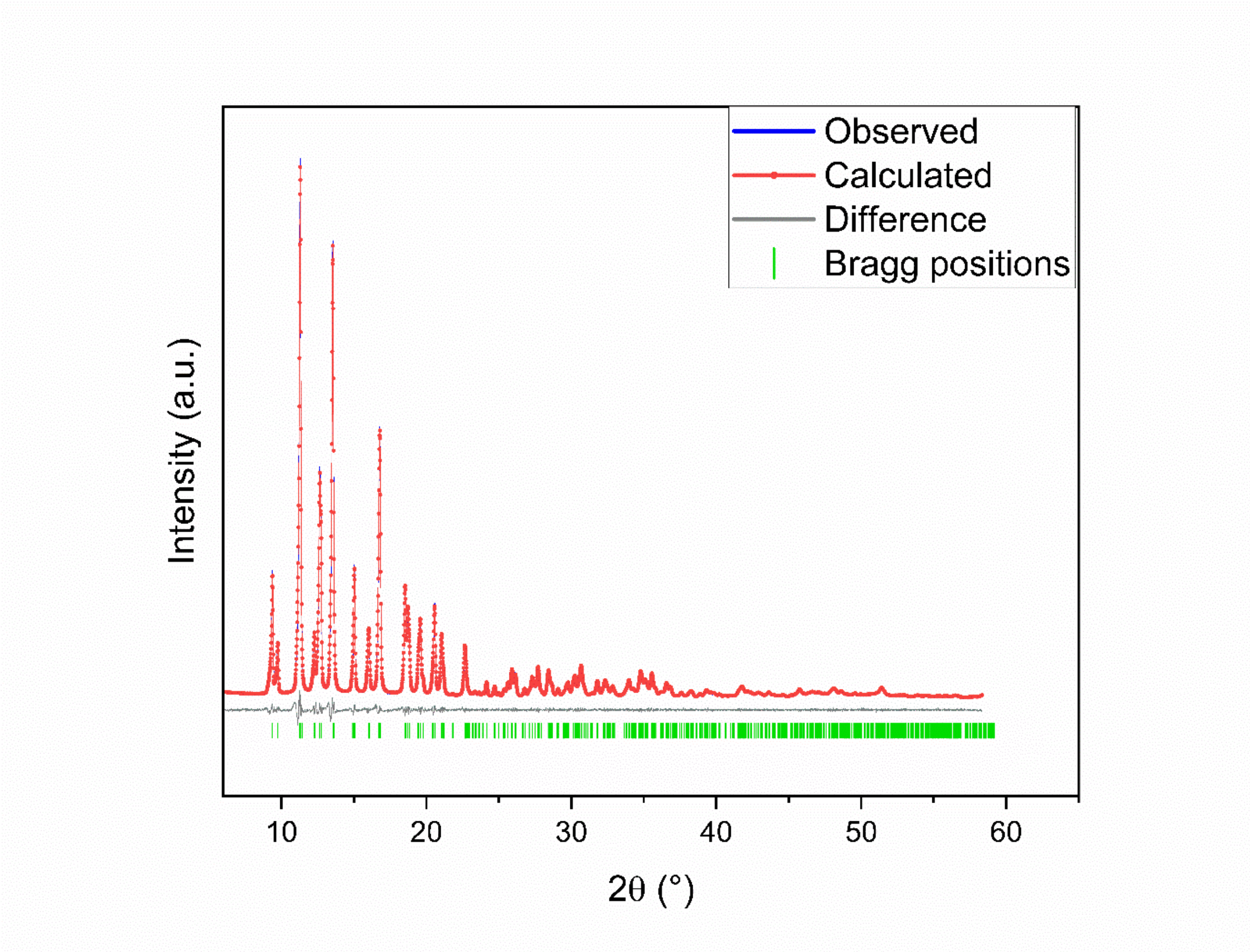

Fig. S1: Pawley refinement of ZIF-62 showing the difference plot (in grey) between the observed and simulated patterns, to confirm sample purity.

Table S5: Pawley refinement data for ZIF-62

R_{wp}	Space Group	Lattice parameters	Lattice Parameters from ZIF-62 CIF[17]
5.609	Pbca	*a* = 15.484160(4) Å<br>*b* = 15.682655(4) Å<br>*c* = 18.095956(4) Å<br>α = 90°<br>β = 90 °<br>γ = 90 °	*a* = 15.5199(2) Å<br>*b* = 15.57710(10) Å<br>*c* = 18.1677(2) Å<br>α = 90 °<br>β = 90 °<br>γ = 90 °

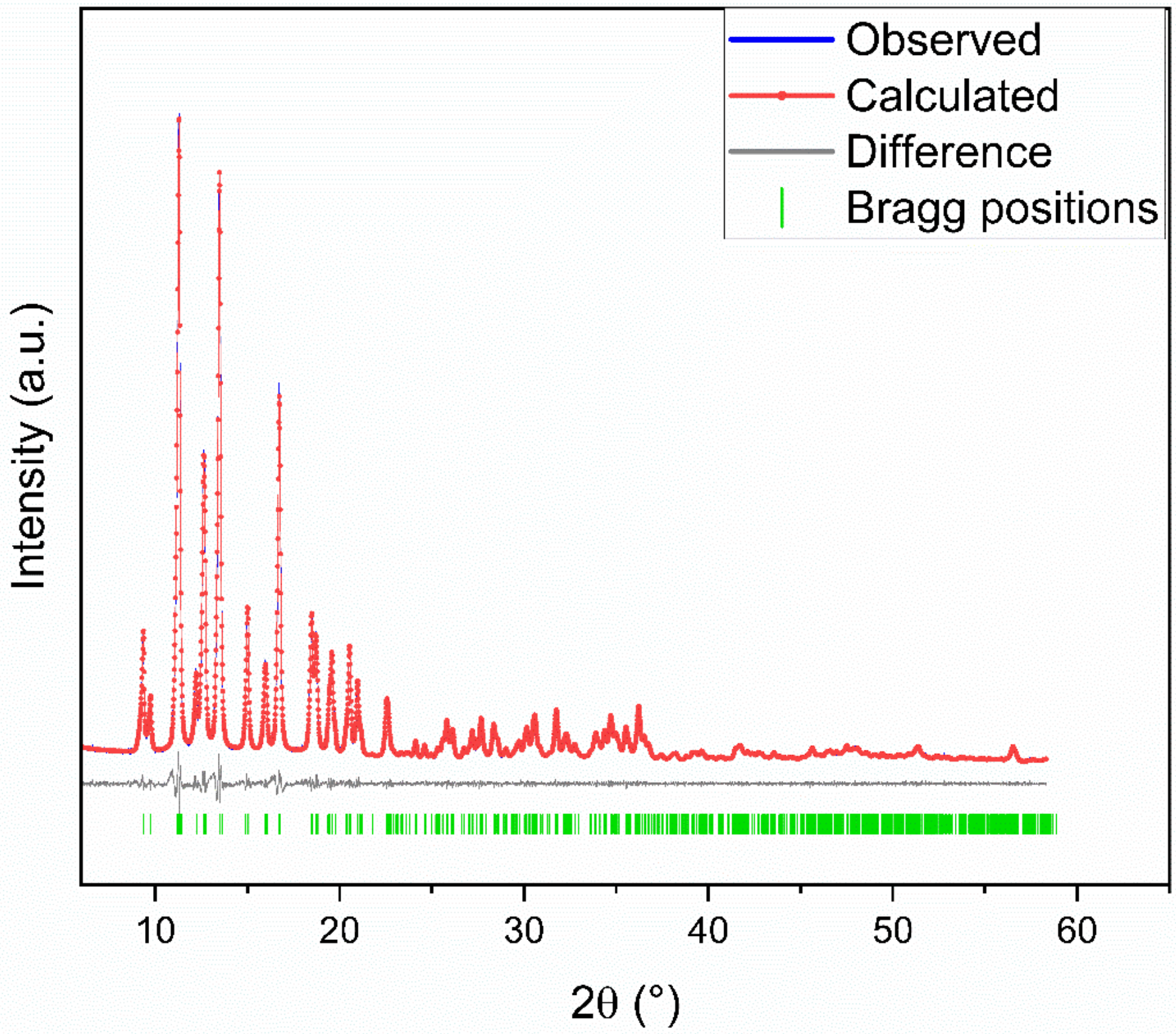

Fig. S2: Pawley refinement of ZIF-62-2mIm$_{0.25}$ showing the difference plot (in grey) between the observed and simulated patterns, to confirm a ZIF-62-like cell.

Table S6: Pawley refinement data for ZIF-62-2mIm$_{0.25}$.

R_{wp}	Space Group	Lattice parameters	Lattice Parameters from ZIF-62-2mIm$_{0.25}$ CIF
5.489	Pbca	*a* = 15. 498079(4) Å<br>*b* = 15. 57284(4) Å<br>*c* = 18. 100403(4) Å<br>α = 90°<br>β = 90 °<br>γ = 90 °	*a* = 15.5199(2) Å<br>*b* = 15.57710(10) Å<br>*c* = 18.1677(2) Å<br>α = 90 °<br>β = 90 °<br>γ = 90 °

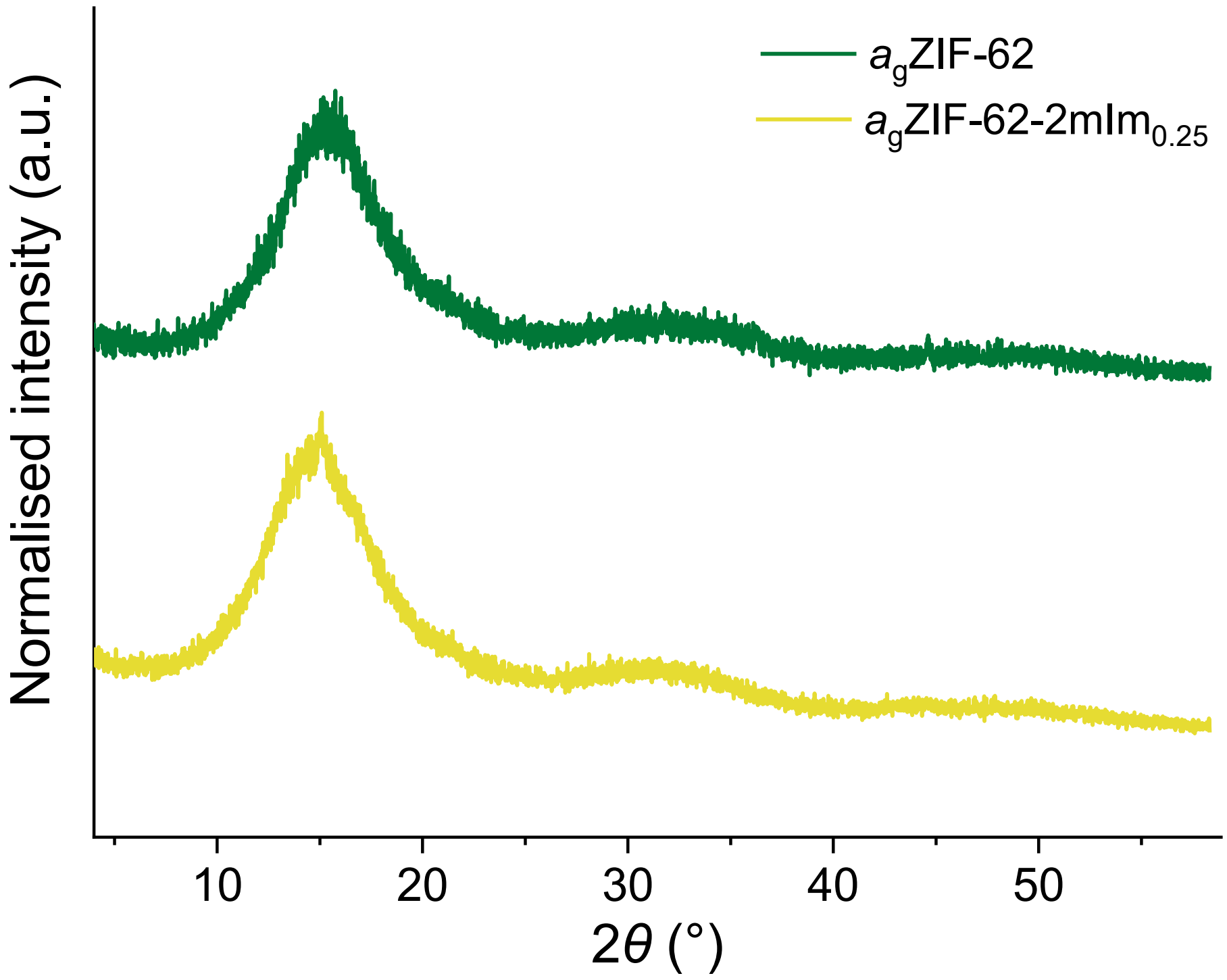

Fig. S3: PXRD of a_gZIF-62 and a_gZIF-62-2mIm$_{0.25}$ displaying broad diffuse peaks characteristic of amorphous materials.

3.2 Single Crystal X-ray Diffraction

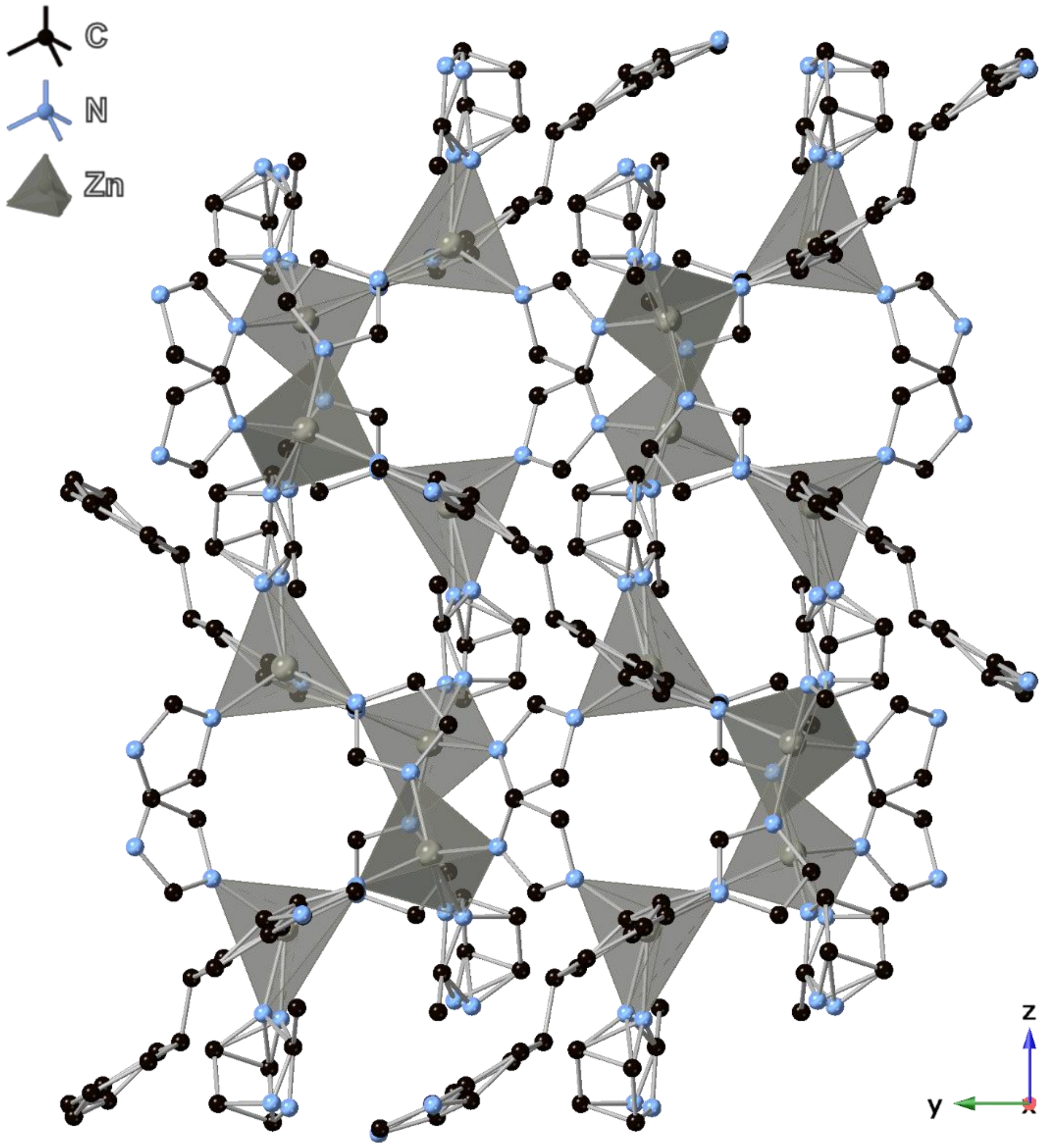

Fig. S4: Crystal structure of SC-ZIF-62-2mIm$_{0.05}$ viewed down the crystallographic *a* axis. Key: Zn tetrahedra in grey, nitrogen in blue and carbon in black (hydrogens omitted for clarity).

Table S7: Crystal data and structure refinement for SC-ZIF-62-2mIm$_{0.05}$

Identification code	bt_al025a_c1_04_sq
Empirical formula	$C_{17.93}H_{19.73}N_{8.85}O_{0.85}Zn_2$
Formula weight / g mol^{-1}	503.56
Temperature/ K	30
Crystal system	orthorhombic
Space group	Pbca
a / Å	15.51986(15)
b / Å	15.57710(14)
c / Å	18.16770(17)
α / °	90
β / °	90
γ / °	90
Volume / Å^3	4392.12(7)
Z	8
ρ_{calc} / g cm^{-3}	1.523
μ / mm^{-1}	2.046
F(000)	2048
Crystal size / mm^3	0.33 × 0.33 × 0.33
Radiation	synchrotron (λ = 0.6889 Å)
2Θ range for data collection / °	4.198 to 51.408
Index ranges	-19 ≤ h ≤ 19, -19 ≤ k ≤ 19, -22 ≤ l ≤ 22
Reflections collected	103530
Independent reflections	4534 [R_{int} = 0.0380, R_{sigma} = 0.0125]
Data/restraints/parameters	4534/521/412
Goodness-of-fit on F^2	1.045
Final R indexes [I>=2σ (I)]	R_1 = 0.0475, wR_2 = 0.1482
Final R indexes [all data]	R_1 = 0.0524, wR_2 = 0.1530
Largest diff. peak/hole / e Å^{-3}	0.56/-0.47

The diffraction data showed significant diffuse scattering even at 30 K, which may be attributed to disordered solvent within the pores as well as disorder of the imidazole substituents (evident in the elongated ellipsoids). The data showed no evidence of twinning or other deficiencies.

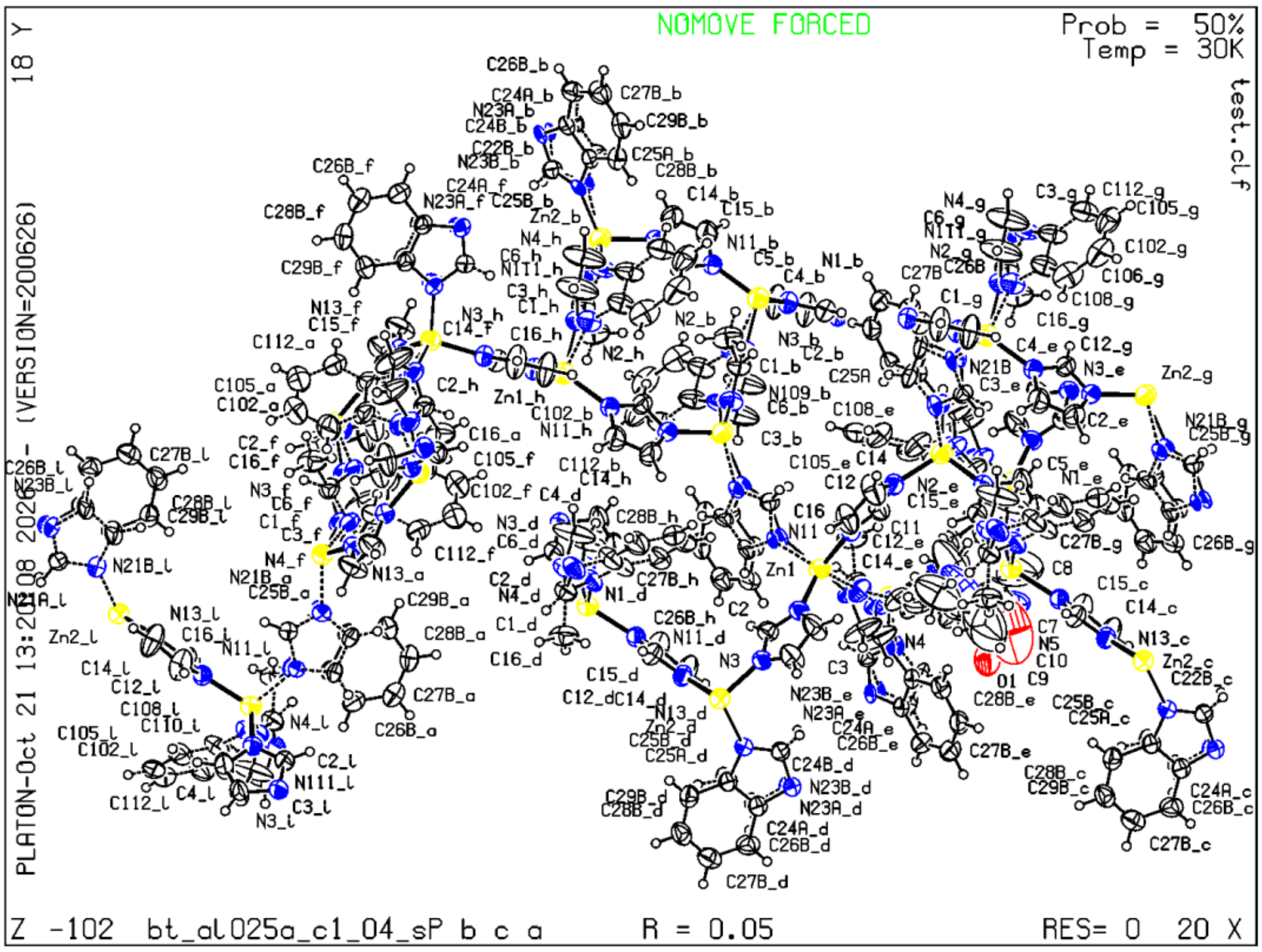

Fig. S5: Thermal ellipsoid plot of SC-ZIF-62-2mIm$_{0.05}$. Ellipsoids are drawn at the 50% probability level.

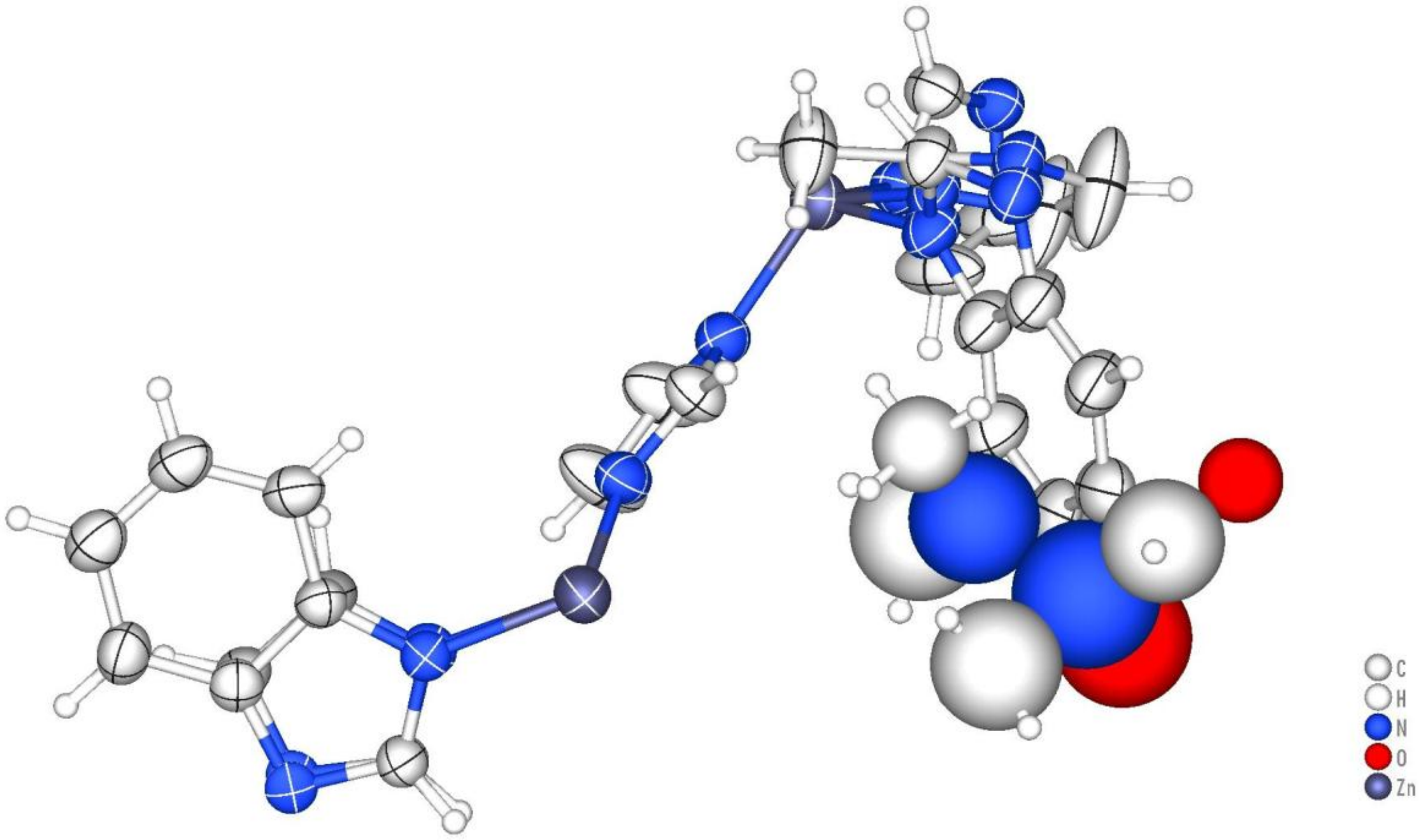

Fig. S6: Thermal ellipsoid plot showing the structural unit including disordered DMF molecules.

3.3 Nuclear Magnetic Resonance (NMR) Spectroscopy

3.3.1 ^{1}H NMR

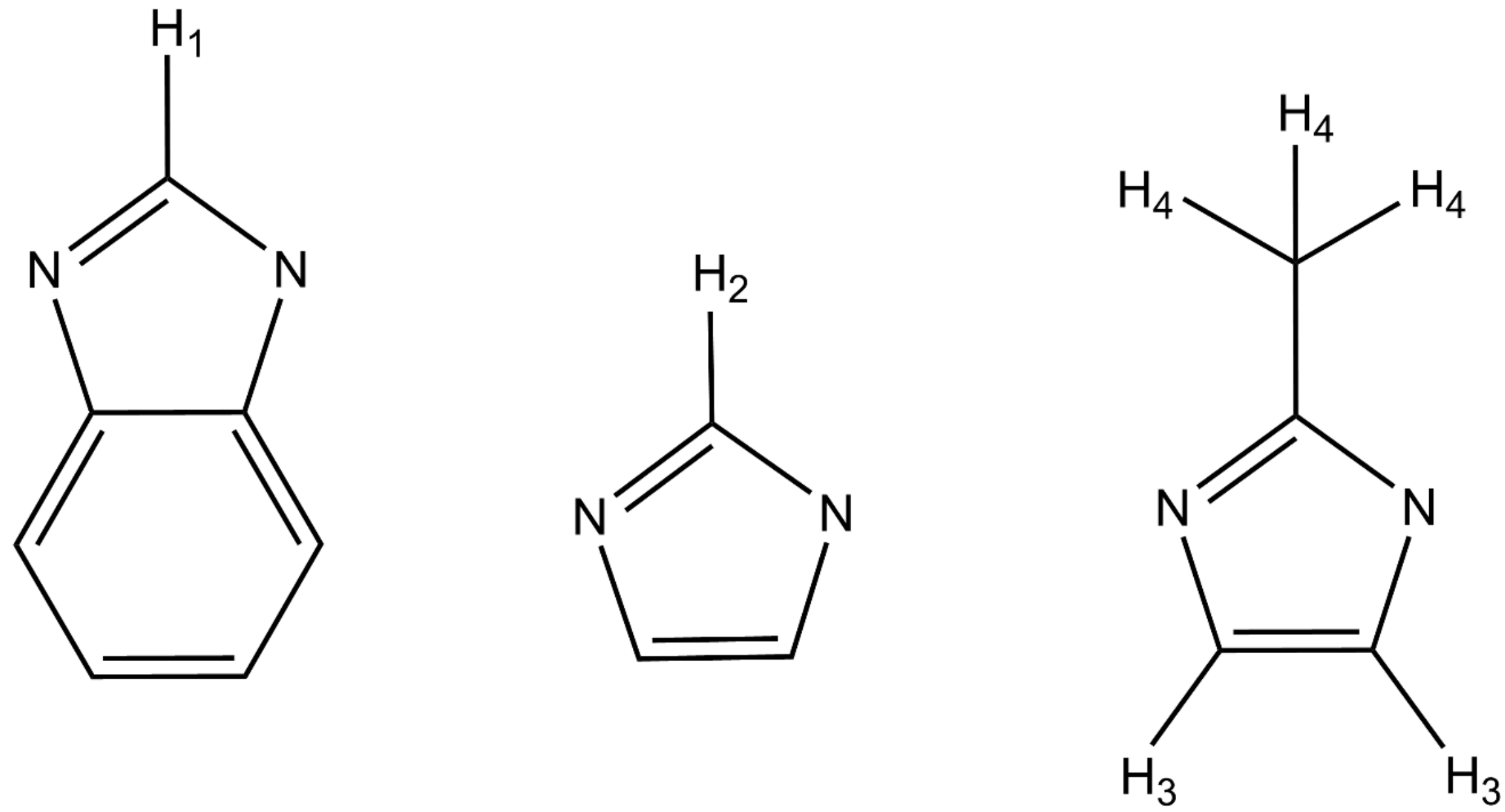

Fig. S7. Structure of Benzimidazole, Imidazole, and 2-methylimidazole, respectively. H_1, H_2, H_3 and H_4 integrations are used to determine linker ratios.

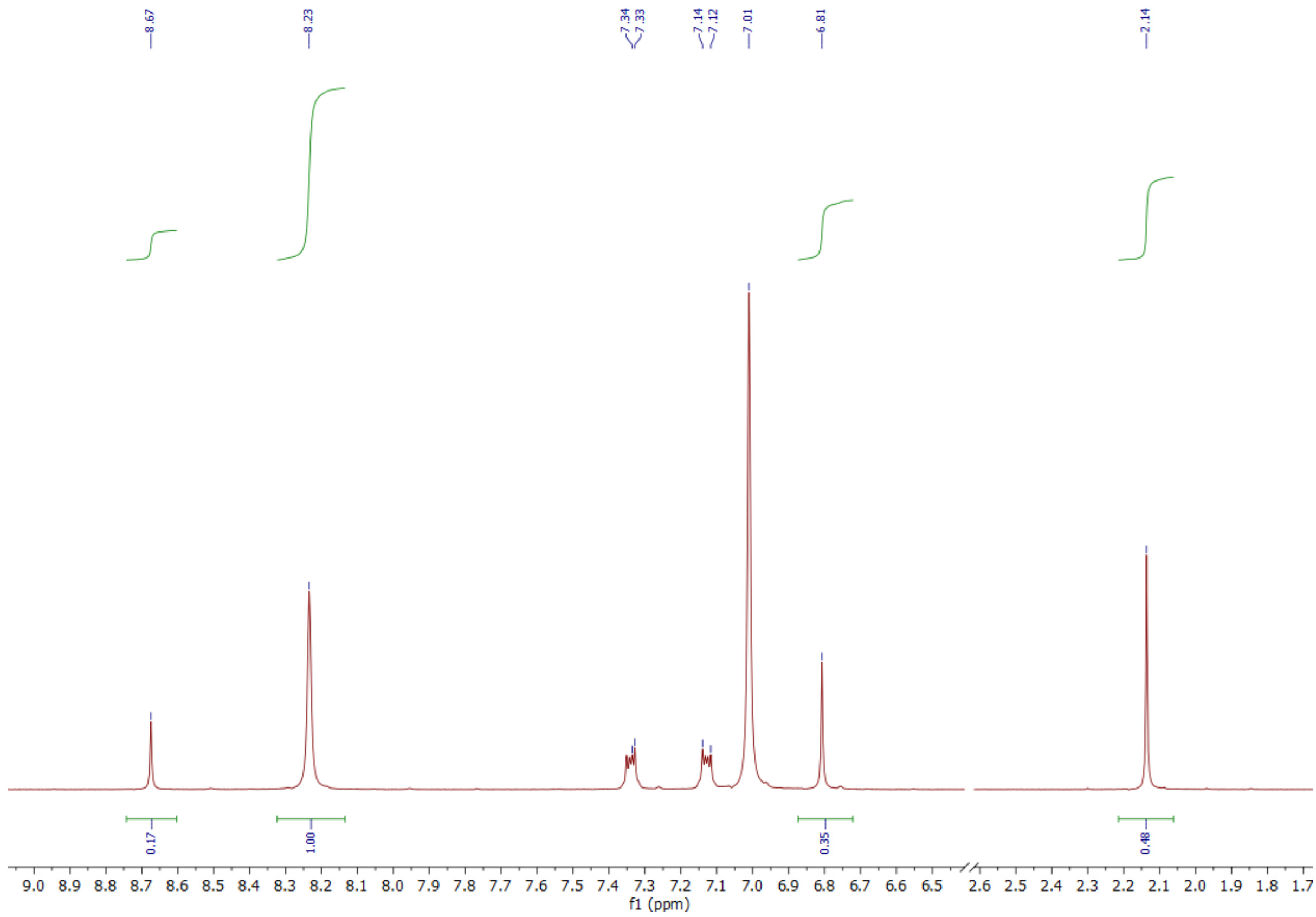

Fig. S8: ^{1}H nuclear magnetic resonance spectra of ZIF-62-2mIm$_{0.25}$ δH(400 MHz; DCl (35%)/D_2O (30 μL) D_2O (600 μL)) 8.67 (1H, s, H_1, bIm), 8.23 (1H, s, H_2, Im), 6.81 (2H, s, H_3, 2-mIm), 2.14 (3H, s, H_4, 2-mIm).

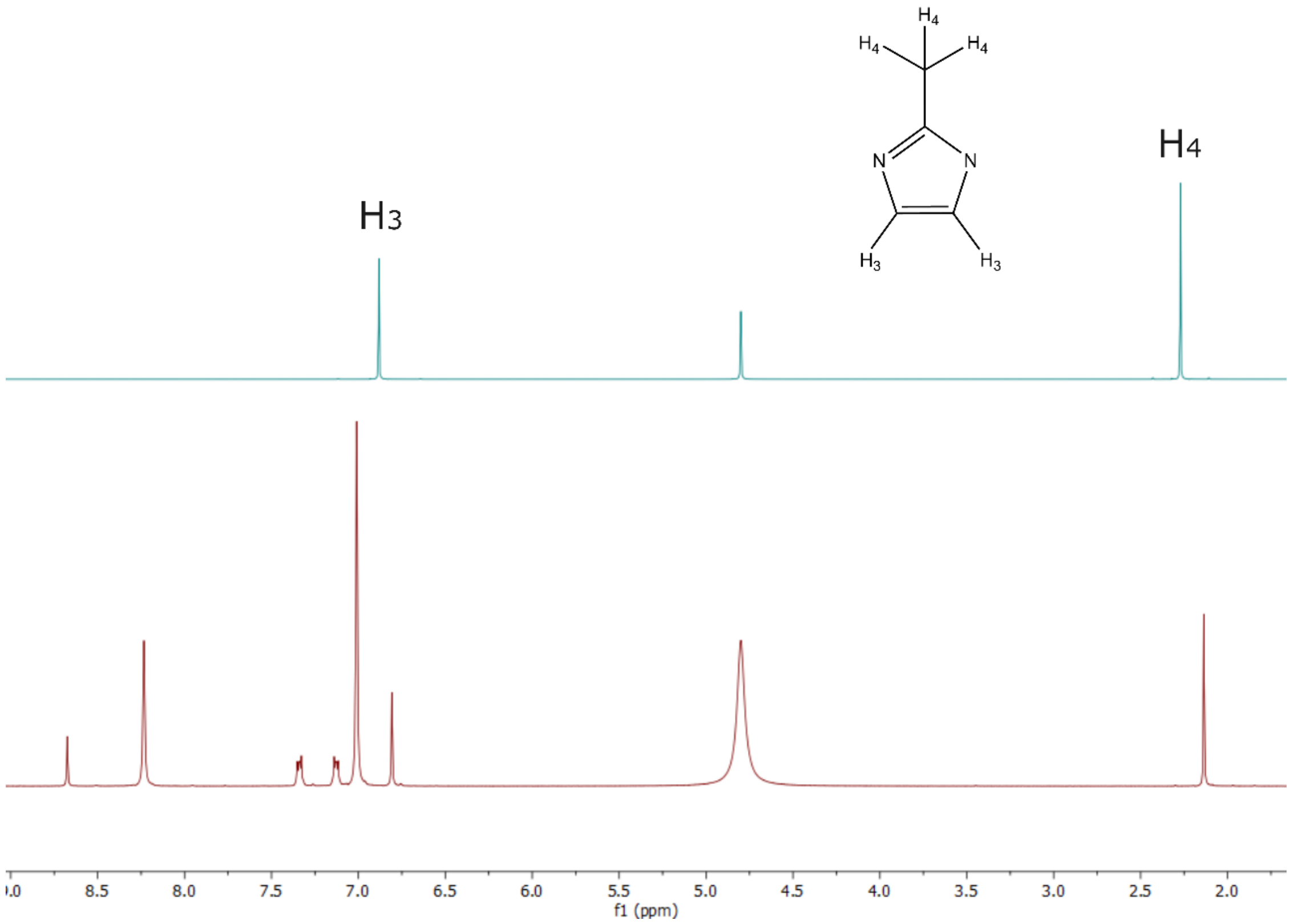

Fig. S9: Stacked ^{1}H nuclear magnetic resonance spectra of ZIF-62-2mIm$_{0.25}$ (red), and 2-methylimidazole (blue), illustrating the 2-methylimidazole peaks present.

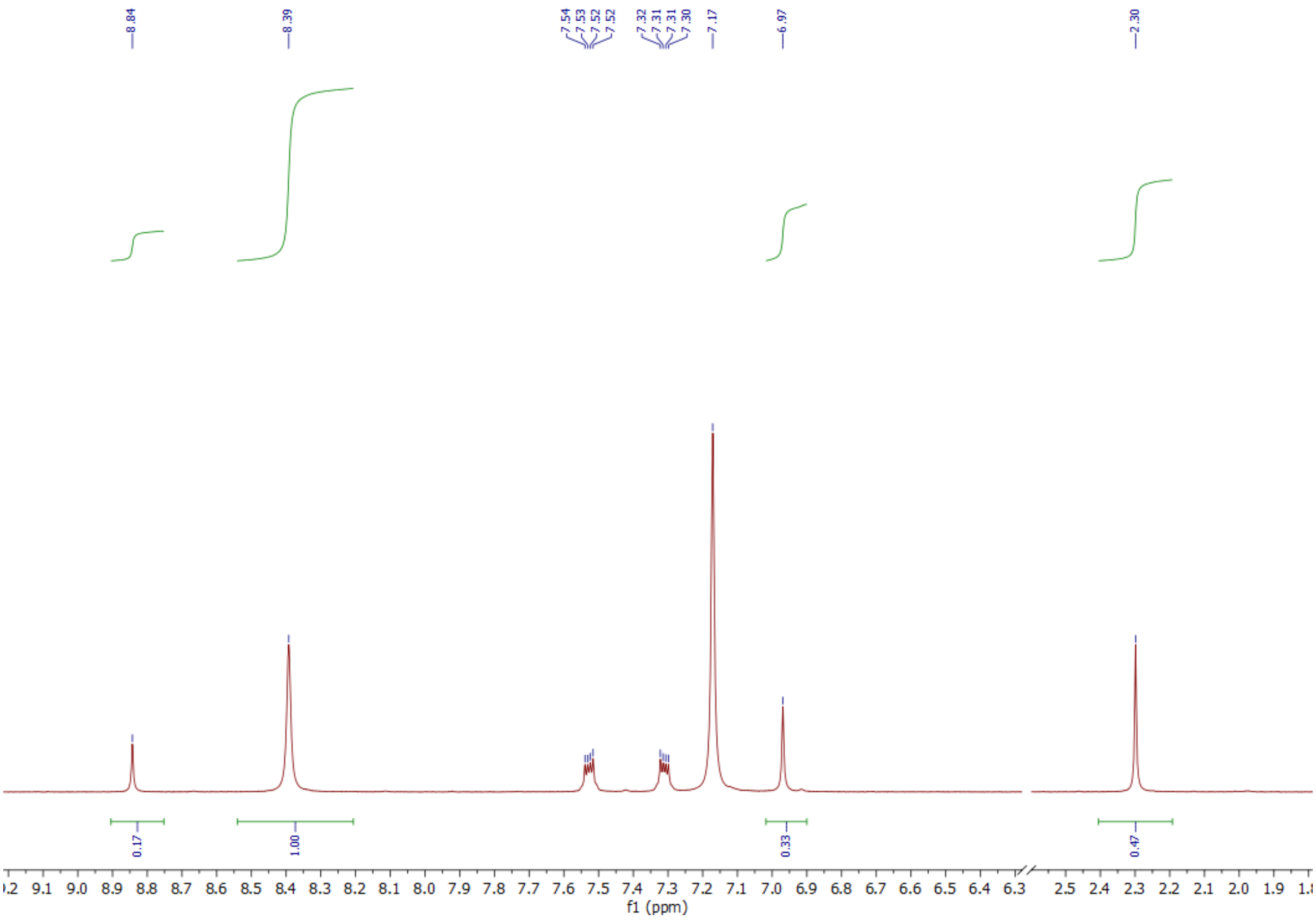

Fig. S10: ^{1}H nuclear magnetic resonance spectra of a_gZIF-62-2mIm$_{0.25}$ δH(400 MHz; DCl (35%)/D_2O (30 μL) D_2O (600 μL)) 8.84 (1H, s, H_1, bIm), 8.39 (1H, s, H_2, Im), 6.97 (2H, s, H_3, 2-mIm), 2.30 (3H, s, H_4, 2-mIm)

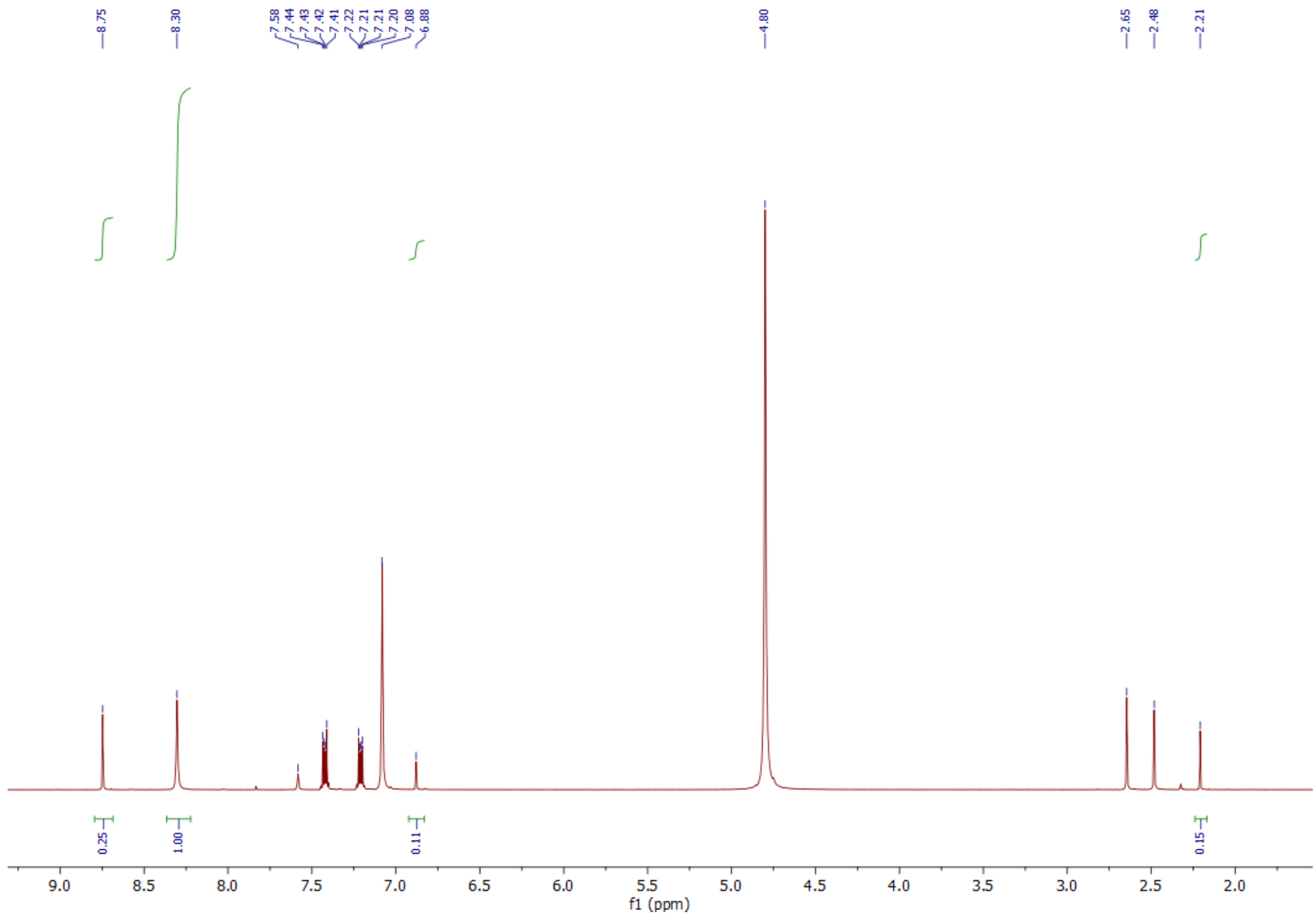

Fig. S11: ^{1}H nuclear magnetic resonance spectra of SC-ZIF-62-2mIm$_{0.05}$ δH(400 MHz; DCl (35%)/D_2O (30 μL) D_2O (600 μL)) 8.75 (1H, s, H_1, bIm), 8.30 (1H, s, H_2, Im), 7.58 (residual DMF), 6.88 (2H, s, H_3, 2-mIm), 2.67, 2.48 (residual DMF), 2.21 (3H, s, H_4, 2-mIm)

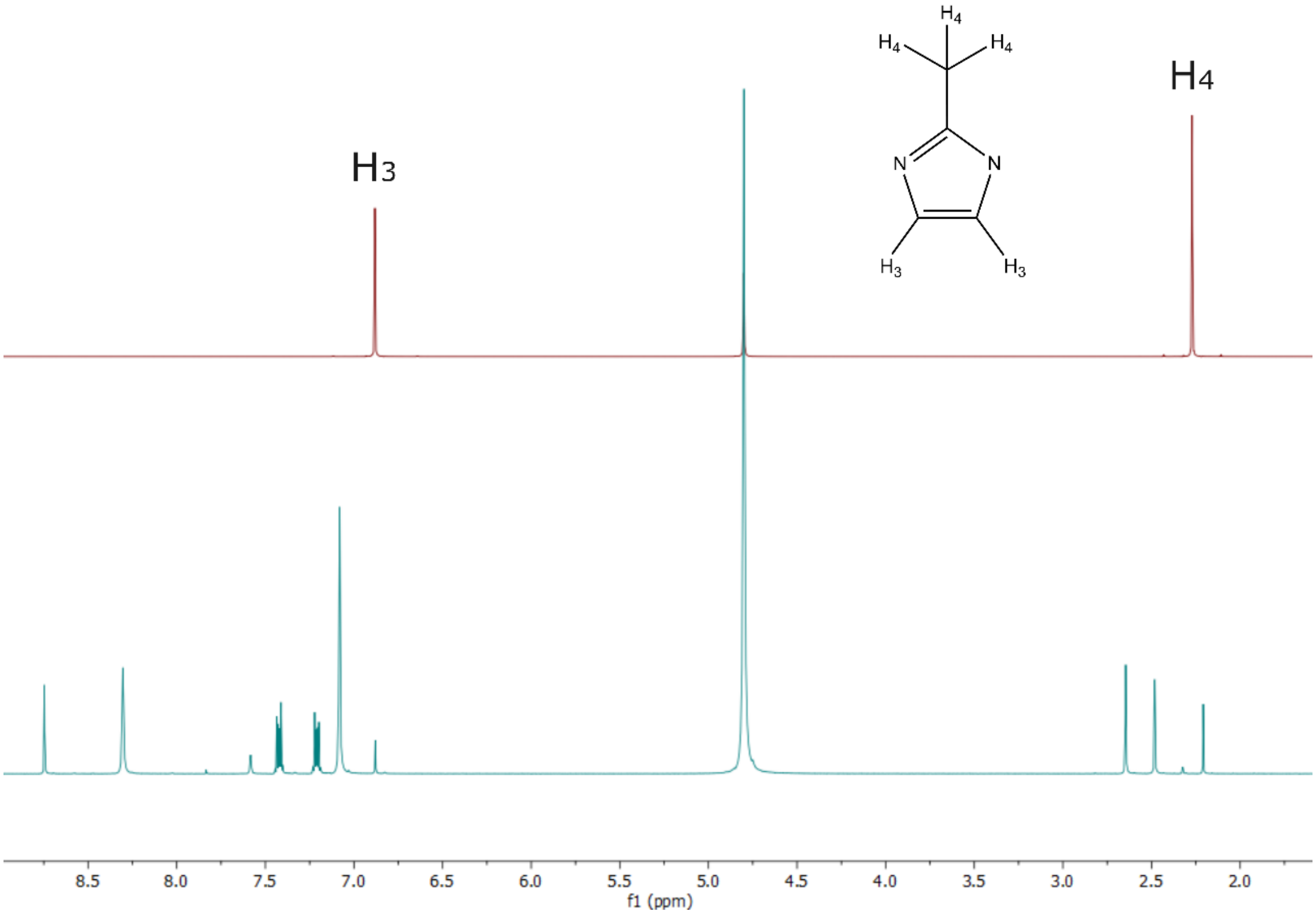

Fig. S12: Stacked ^{1}H nuclear magnetic resonance spectra of SC-ZIF-62-2mIm$_{0.05}$(blue) and 2-methylimidazole (red), illustrating the 2-methylimidazole peaks present.

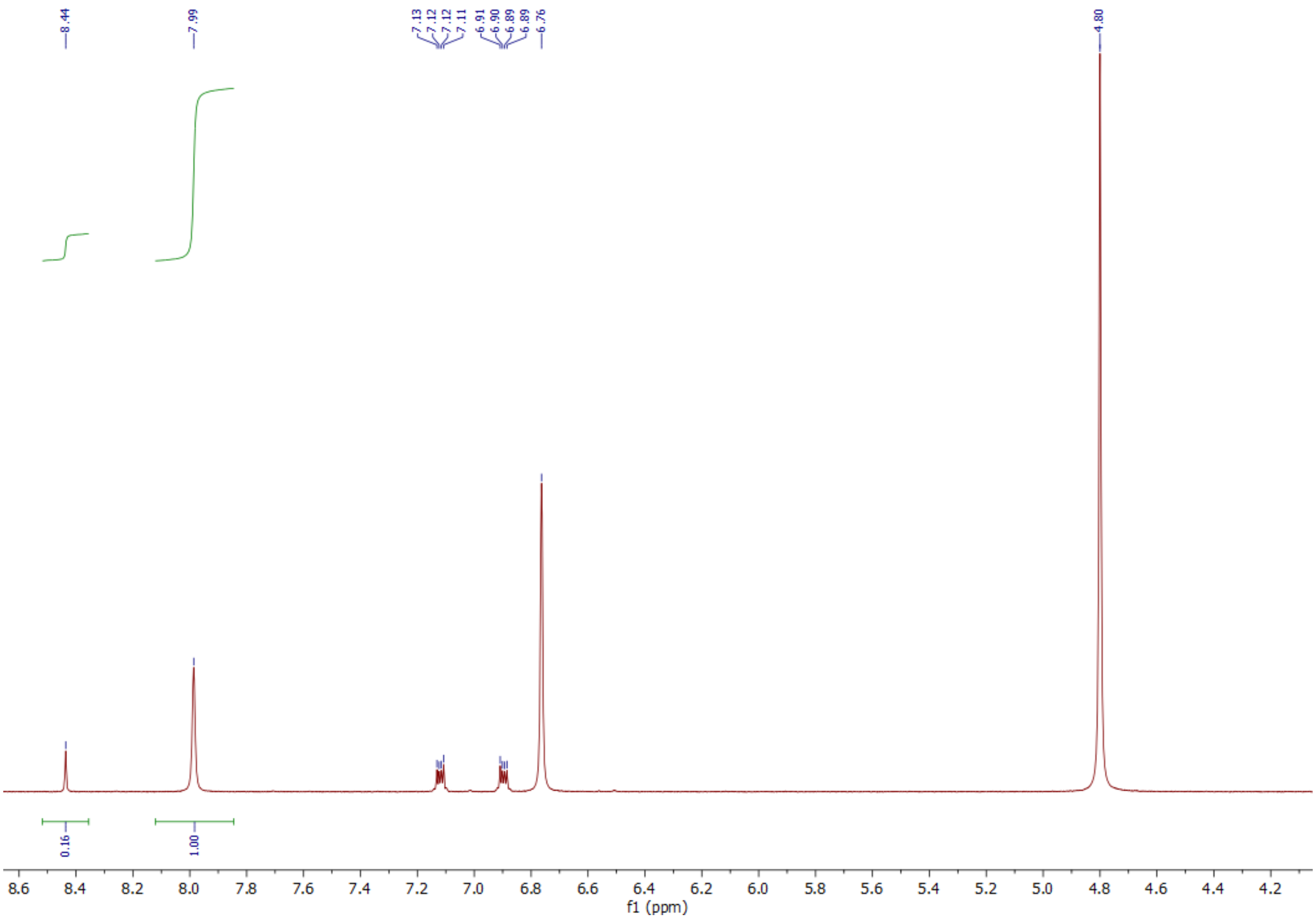

Fig. S13: ^{1}H nuclear magnetic resonance spectra of ZIF-62 δH(400 MHz; DCl (35%)/D_2O (30 μL) D_2O (600 μL)) 8.44 (1H, s, H_1, bIm), 7.99 (1H, s, H_2, Im), 4.8 (D_2O).

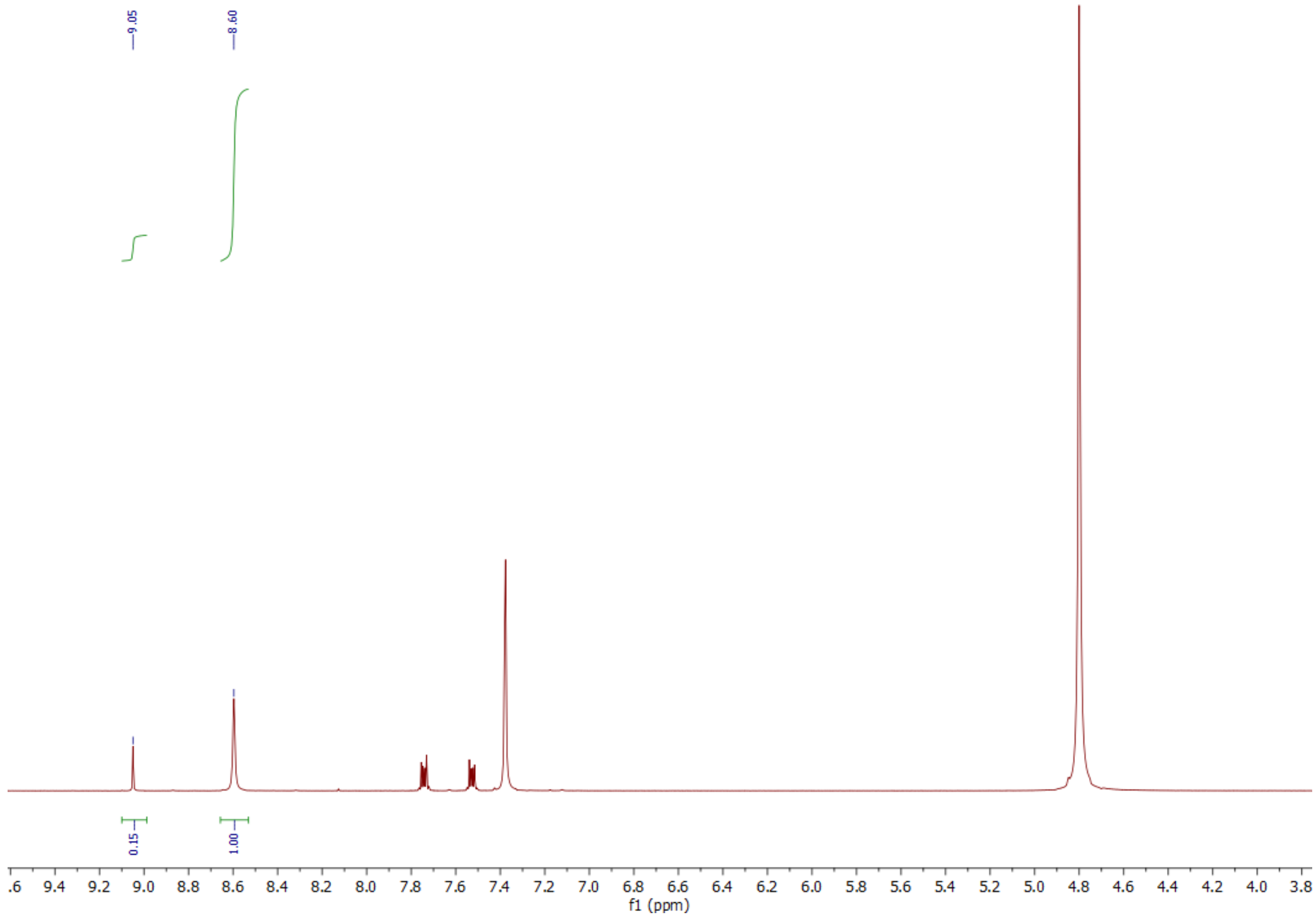

Fig. S14: ^{1}H nuclear magnetic resonance spectra of a_gZIF-62 δH(400 MHz; DCl (35%)/D_2O (30 μL) D_2O (600 μL)) 9.05 (1H, s, H_1, bIm), 8.60 (1H, s, H_2, Im), 4.8 (D_2O).

3.3.2 Solid-state NMR

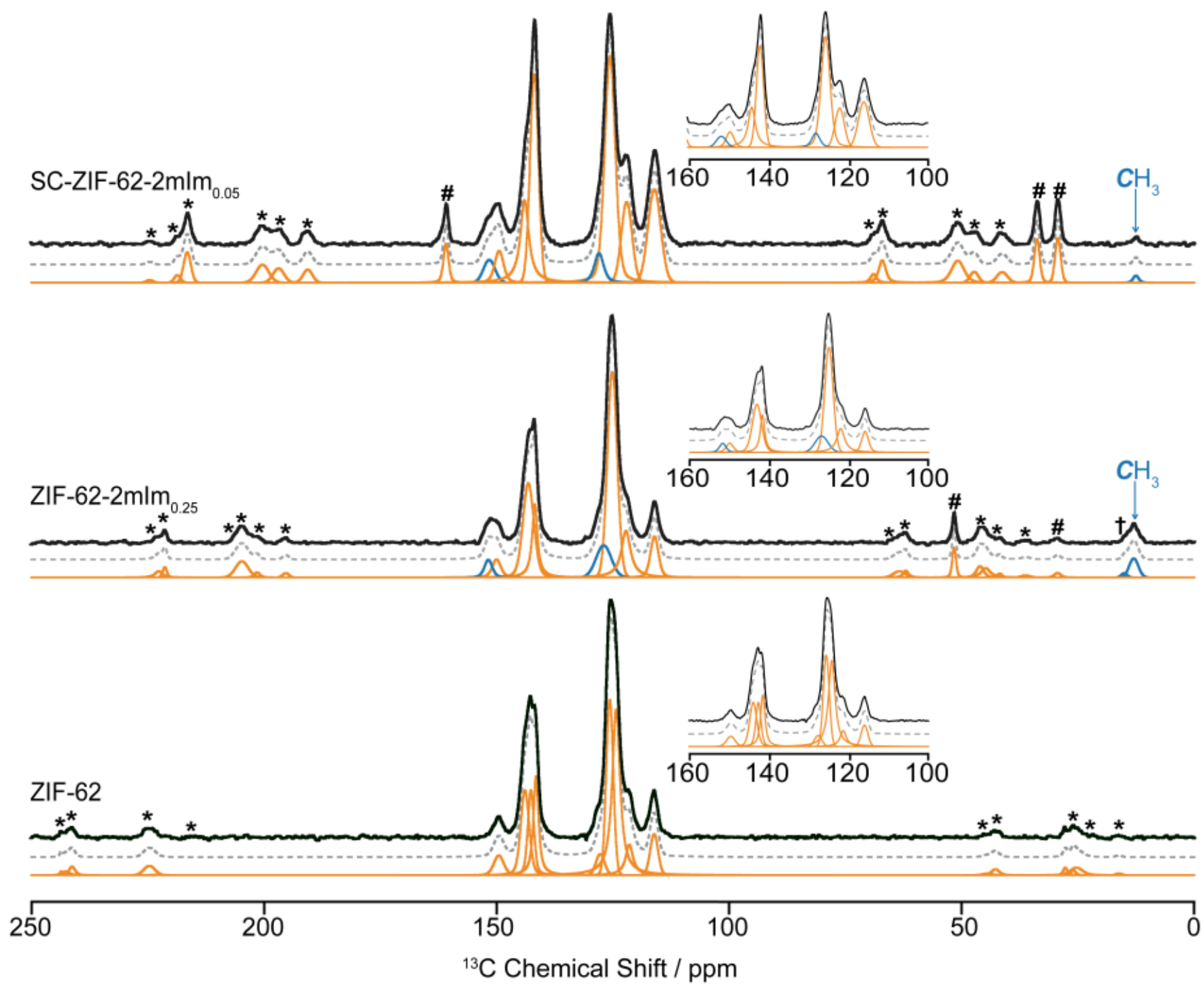

Fig. S15: Directly excited ^{13}C MAS NMR spectra of ZIFs. Experimental spectrum (black line), total fit (dashed grey line) and spectral deconvolution (orange and blue lines) are shown. The signals assigned as unique environments for 2-mIm are labelled in blue. Inserts show the deconvolution of sites in the region 100-160 ppm. The MAS rates used are 7.50, 8.0 and 10.0 kHz for SC-ZIF-62-2mIm$_{0.05}$, ZIF-62-2mIm$_{0.25}$ and ZIF-62, respectively as given in Table S8. Assignments given in Table S10. Spinning sidebands, identified from spectra at variable MAS (Fig. S18) are marked with asterisks (*), the 2-mIm shoulder is marked with a dagger (†), hashtags (#) show signals from residual solvents (DMF at 160.6, 33.6, and 29.6 ppm or DCM at 51.9 ppm).

Table S8: Additional acquisition parameters for directly excited ^{13}C MAS NMR spectra (Fig.S15). Full experimental details are given in experimental section.

Sample	MAS rate / kHz	Recycle delay / s
SC-ZIF-62-2mIm$_{0.05}$	7.5	90.0
ZIF-62-2mIm$_{0.25}$	8.0	50.0
ZIF-62	10.0	46.0

MAS rates of ^{13}C direct excitation spectra differed as they were chosen to avoid overlapping sidebands, solvents and methyl peaks. Chemical shifts for ZIF-62 were in good agreement with those previously reported.[15] Deconvolution of ZIF-62 contained additional sites beyond the expected total number of six environments for Im and bIm in ZIF-62. This likely represents two sets of Im sites due to their presence in distinct tetrahedra which either contain four imidazole linkers or three imidazole and one benzimidazole linker, causing a slight change in chemical shift (such as N***C***N at 143.9 and 142.6 ppm). Similar fits have been reported for ZIF-62 by Qiao *et al* who

suggested certain environments on bIm would also present multiple unique resonances given the possibility of tetrahedra containing more than one benzimidazole linker.[16] The stoichiometry for ZIF-62 was determined to be $(Im)_{1.75}(bIm)_{0.25}$, in agreement with its nominal composition.

Similarly, within the deconvolution of the ZIF-62-2mIm$_{0.25}$ spectrum the peak corresponding to the methyl group of 2-mIm was fitted with 2 components at 14.8 and 13.2 ppm. This was done due to the presence of a small shoulder, that is independent of MAS (Fig. S18b), which is most likely due to the presence of 2-mIm within multiple distinct tetrahedra. Furthermore, although the chemical shift of this minor component aligns closely with pristine 2-mIm (Fig. S17) it is unlikely that this is residual linker as this is not observed via PXRD (Fig.1 & S2). Moreover, the presence of any residual linker would likely severely hinder gas uptake measurements and this was not the case.

The stoichiometry of ZIF-62-2mIm$_{0.25}$ was determined to be $(Im)_{1.60}(2\text{-}mIm)_{0.21}(bIm)_{0.19}$, showing a near 1:1 ratio between 2-mIm and bIm in agreement with the nominal composition of $(Im)_{1.50}(2\text{-}mIm)_{0.25}(bIm)_{0.25}$ whereas SC-ZIF-62-2mIm$_{0.05}$ contained a linker ratio of $(Im)_{1.30}(2\text{-}mIm)_{0.05}(bIm)_{0.65}$.

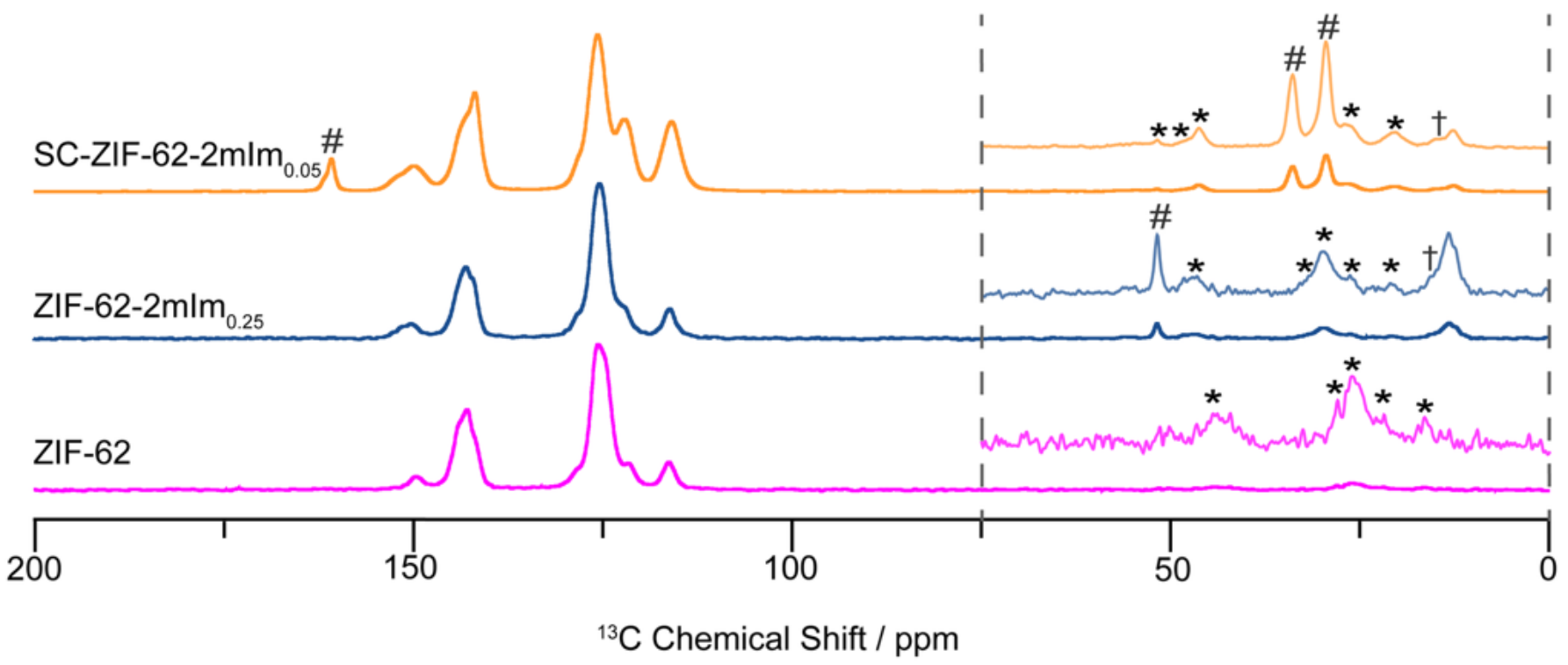

Fig. S16: ^{13}C CP MAS NMR spectra of both SC-ZIF-62-2mIm$_{0.05}$ and ZIF-62-2mIm$_{0.25}$ samples recorded with a contact time of 0.5 ms with ZIF-62 for comparison. Additional acquisition parameters given in Table S9. Assignments given in Table S10. Inserts show magnified views of the spectra in the 75 to 0 ppm region. Spinning sidebands, identified from spectra at variable MAS (Fig. S18) are marked with asterisks (*), dagger (†) shows the 2-mIm shoulder, hashtags (#) show signals from residual solvents (DMF at 160.6, 33.6, and 29.6 ppm or DCM at 51.9 ppm).

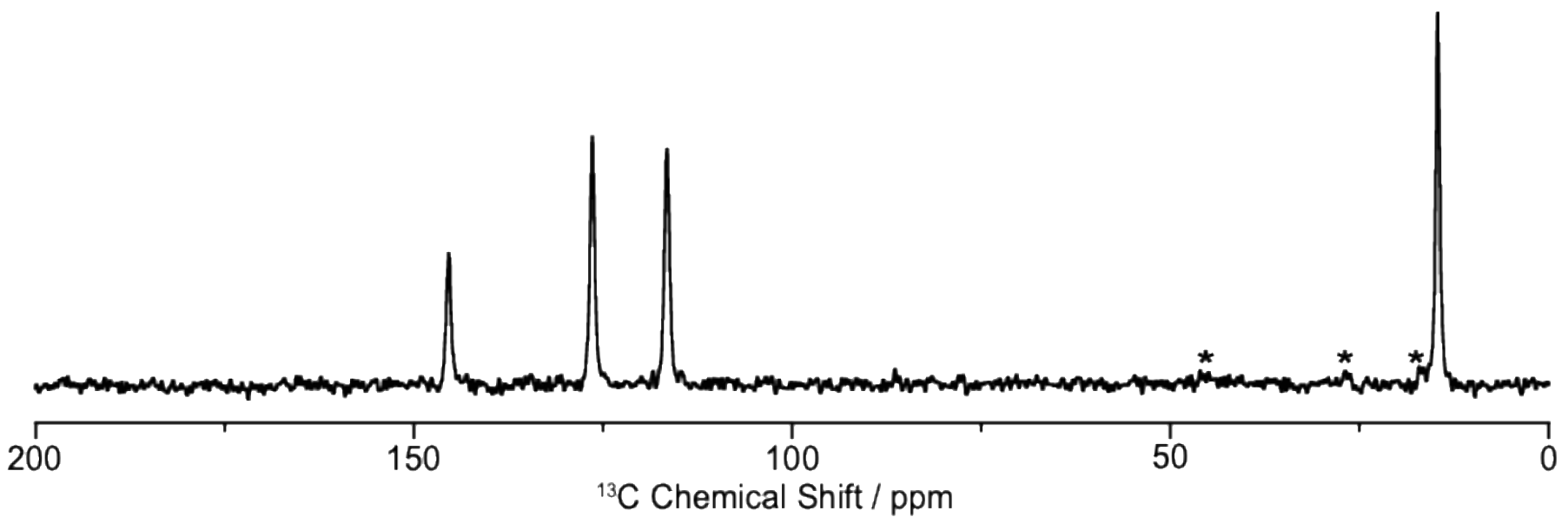

Fig. S17: ^{13}C CP MAS NMR spectrum of 2-mIm recorded with a contact time of 0.5 ms under a MAS frequency of 10 kHz. Assignments given in Table S10. Spinning sidebands are marked with asterisks (*).

Table S9: Additional acquisition parameters for ^{13}C CP MAS NMR spectra acquired with a contact time of 0.5 ms (Figs. S16 & S17).

Sample	MAS rate / kHz	Recycle delay / s
SC-ZIF-62-2mIm$_{0.05}$	9.6	3.0
ZIF-62-2mIm$_{0.25}$	9.6	3.0
ZIF-62	10.0	3.0
2-mIm	10.0	3.0

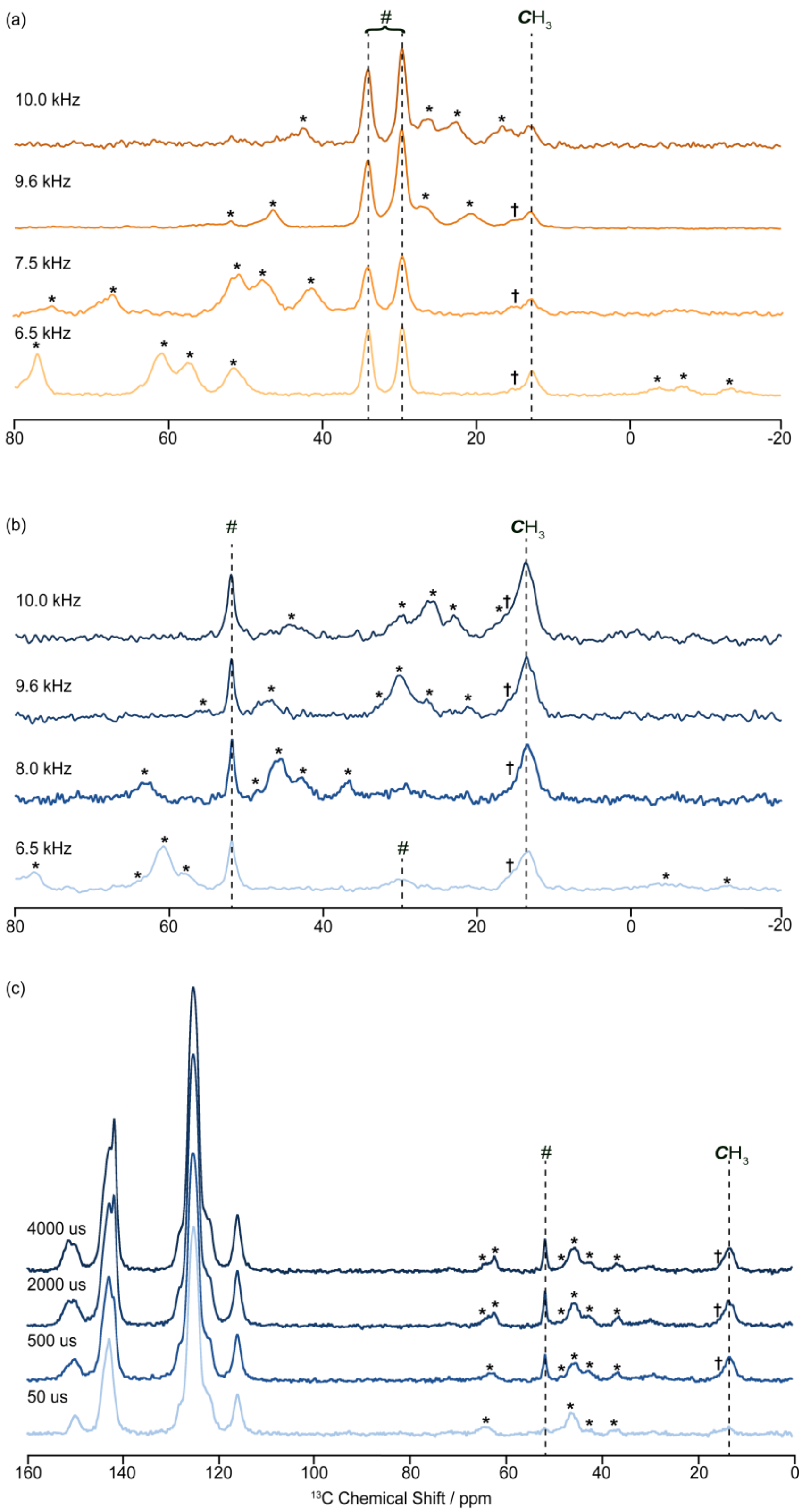

Fig. S18: ^{13}C CP MAS spectra of (a) SC-ZIF-62-2mIm$_{0.05}$ and (b) ZIF-62-2mIm$_{0.25}$, with varied MAS frequencies, and of (c) ZIF-62-2mIm$_{0.25}$ recorded with differing contact times under MAS frequency of 8 kHz. Spinning sidebands, are marked with asterisks (*), the 2-mIm shoulder is marked with a dagger (†), hashtags (#) show signals from residual solvents (DMF at 160.6, 33.6, and 29.6 ppm or DCM at 51.9 ppm).

Table S10: ^{13}C chemical shifts with proposed spectral assignments for SC-ZIF-62-2mIm$_{0.05}$, ZIF-62-2mIm$_{0.25}$, ZIF-62 and 2-mIm.

ZIF Sample	^{13}C Chemical Shift / ppm	Assignment
SC-ZIF-62-2mIm$_{0.05}$	160.6	***C***O (DMF)
	151.8, 149.9	N***C***N (2-mIm), N***C***N (bIm)
	143.2	N***C***N (Im)
	141.7	CH***C***N (bIm)
	128.0, 125.5	N***C***C (2-mIm), N***C***C (Im)
	122.1	***C***HCHCN (bIm)
	115.8	CH***C***HCN (bIm)
	33.8	***C***H$_3$ (DMF)
	29.4	***C***H$_3$ (DMF)
	14.3	***C***H$_3$ (2-mIm)
	12.9	***C***H$_3$ (2-mIm)
ZIF-62-2mIm$_{0.25}$	151.7, 149.9	N***C***N (2-mIm), N***C***N (bIm)
	143.2	NCN (Im)
	141.8	CH**C**N (bIm)
	128.0, 125.3	N**C**C (2-mIm), N**C**C (Im)
	122.2	***C***HCHCN (bIm)
	116.1	CH***C***HCN (bIm)
	51.8	***C***H$_2$Cl$_2$ (DCM)
	29.6	***C***H$_3$ (DMF)
	14.8	***C***H$_3$ (2-mIm)
	13.2	***C***H$_3$ (2-mIm)
ZIF-62	149.5	N***C***N (bIm)
	143.9, 142.6	N***C***N (Im)
	141.6	CH***C***N (bIm)
	127.7, 125.7, 124.4	N***C***C (Im)
	121.5	***C***HCHCN (bIm)
	116.2	CH***C***HCN (bIm)
2-mIm	144.8	N***C***N
	125.8	N***C***C
	116.0	NH***C***C
	14.2	***C***H$_3$

3.4 Differential Scanning Calorimetry

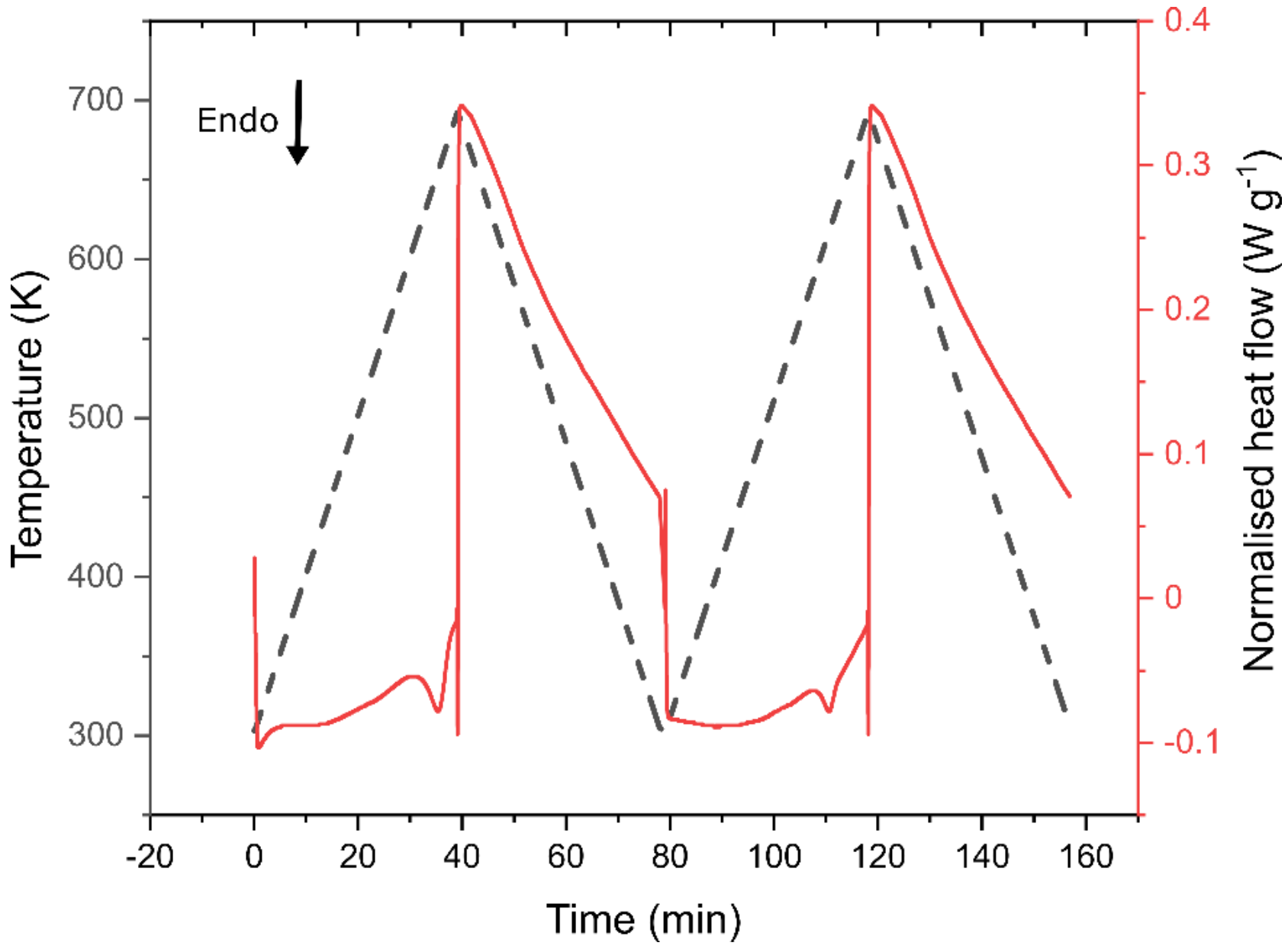

Fig. S19: DSC trace of [Zn(Im)$_{1.75}$(bIm)$_{0.25}$] showing the temperature (Black dashed line) ramping method from 303.15 K to 693.15 K repeatedly, and the thermal events observed (red line).

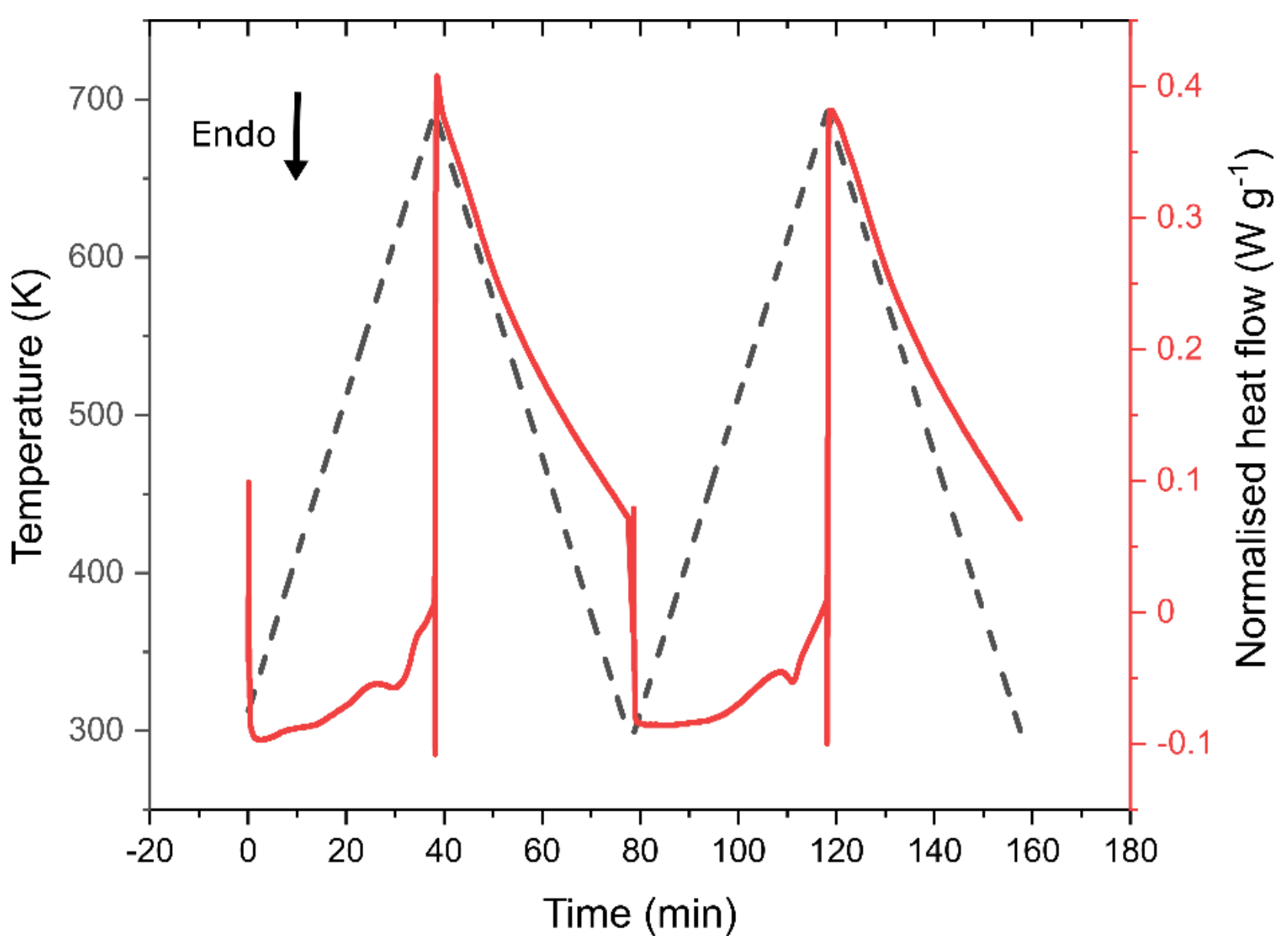

Fig. S20: DSC trace of [Zn(Im)$_{1.50}$(2-mIm)$_{0.25}$(bIm)$_{0.25}$] showing the temperature (black dashed line) ramping method from 303.15 K to 693.15 K repeatedly, and the thermal events observed (red line).

3.5 Pair distribution function (PDF)

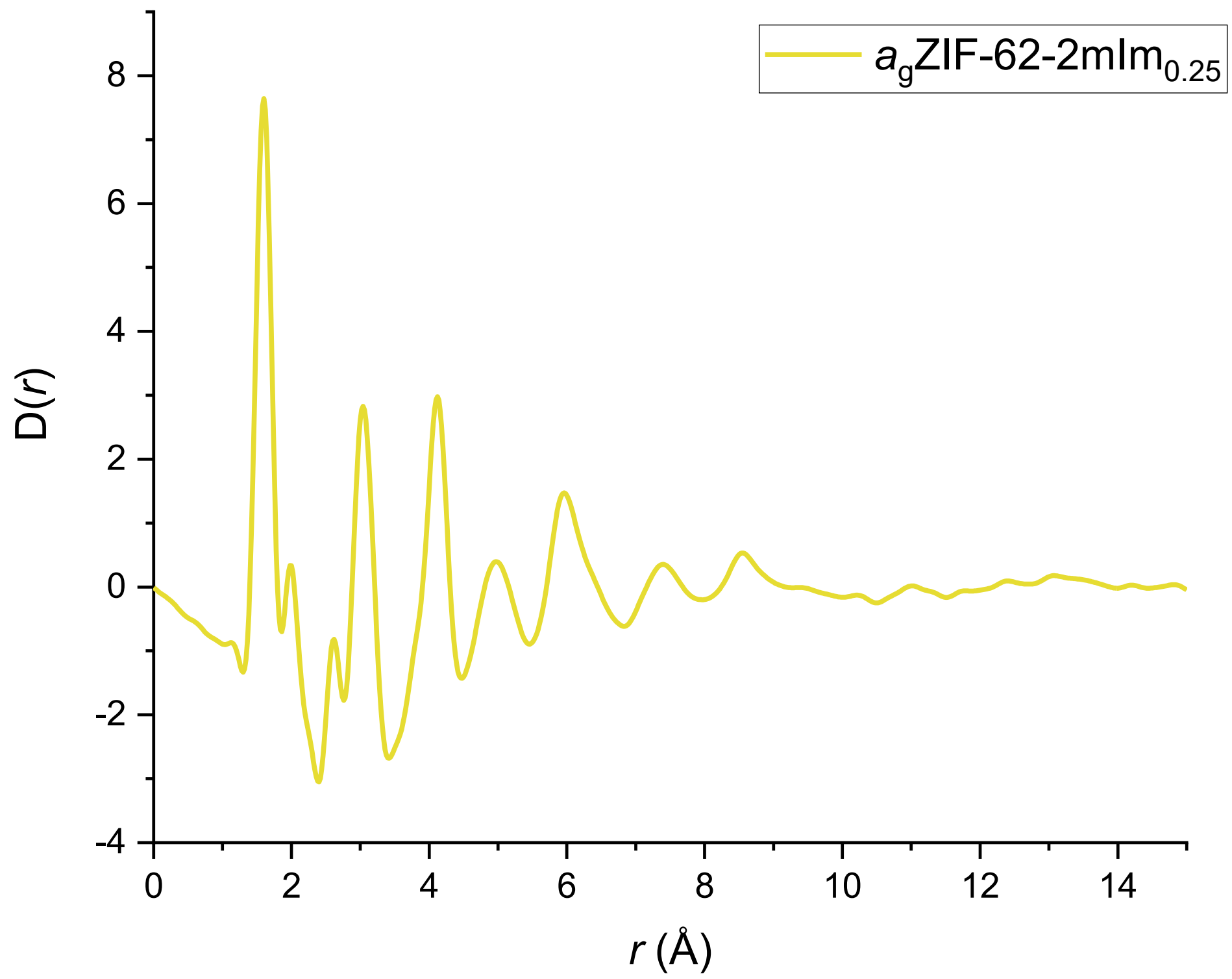

Fig. S21: D(*r*) for a_gZIF-62-2mIm$_{0.25}$, correlations between 1 and 8 Å correspond to the local structure of the Zn-2-mIm-Zn unit.

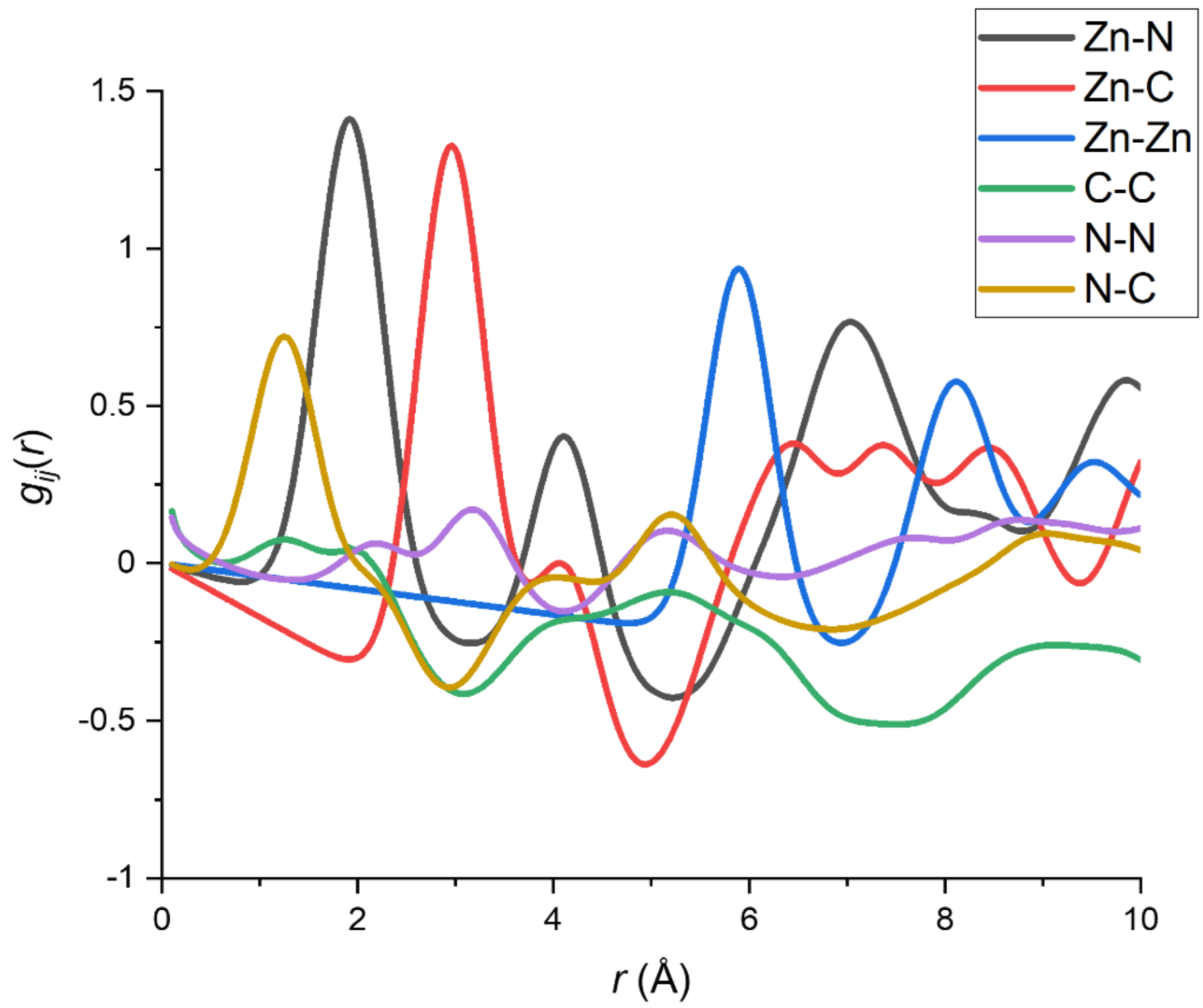

Fig. S22: gij(r) of ZIF-62-2mIm$_{0.25}$ illustrating the individual correlations that contribute to the overall D(r). These gij(r) were calculated from the CIF file collected for ZIF-62-2mIm$_{0.25}$.

Table S11: Partial PDF assignments for a_gZIF-62-2mIm$_{0.25}$

ZIF Sample	r / Å	Assignment
a_gZIF-62-2mIm$_{0.25}$	1.6	N-C, C-C
	2	Zn-N, C-C
	2.6, 3	Zn-C, aromatic and methyl
	4.1	Zn-N
	5	N-C
	6	Zn-Zn
	7.4	Zn-N

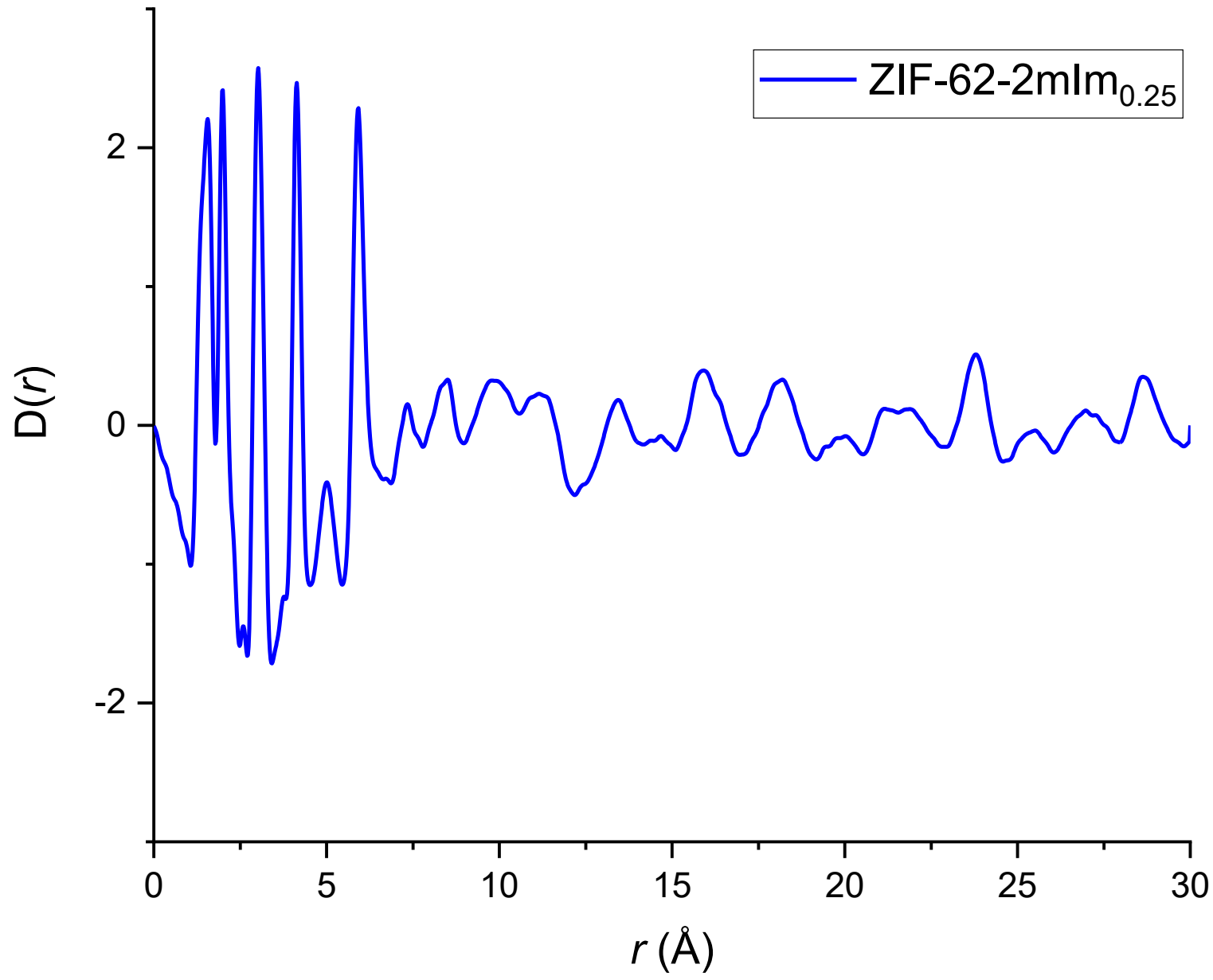

Fig. S23: D(r) for ZIF-62-2mIm$_{0.25}$, correlations between 1 and 8 Å correspond to the local structure of the Zn-2-mIm-Zn unit, while correlations beyond 8 Å correlate to the long-range order expected in a crystalline structure.

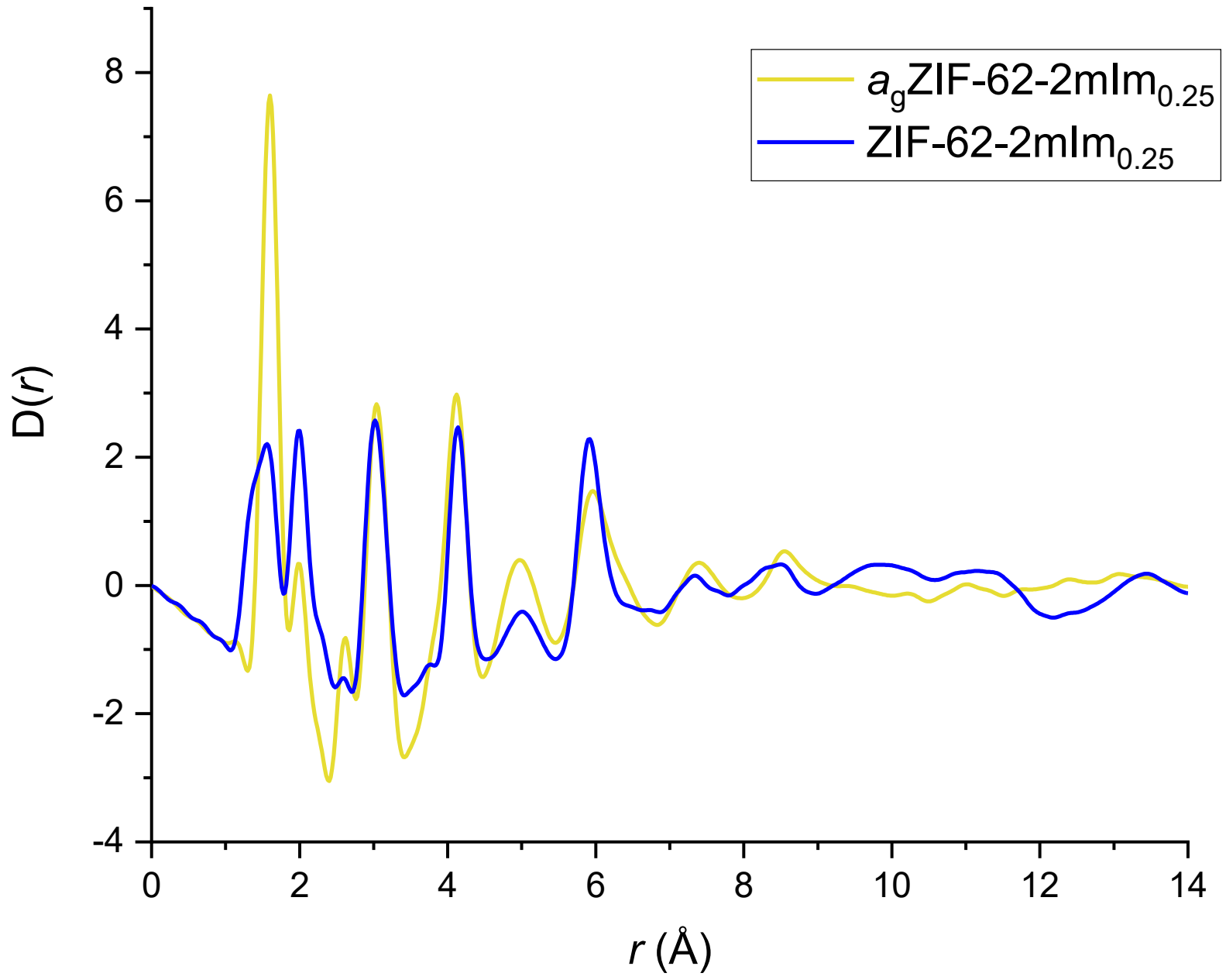

Fig. S24: D(r) for ZIF-62-2mIm$_{0.25}$ and a_gZIF-62-2mIm$_{0.25}$, correlations between 1 and 8 Å correspond to the local structure of the Zn-2-mIm-Zn unit.

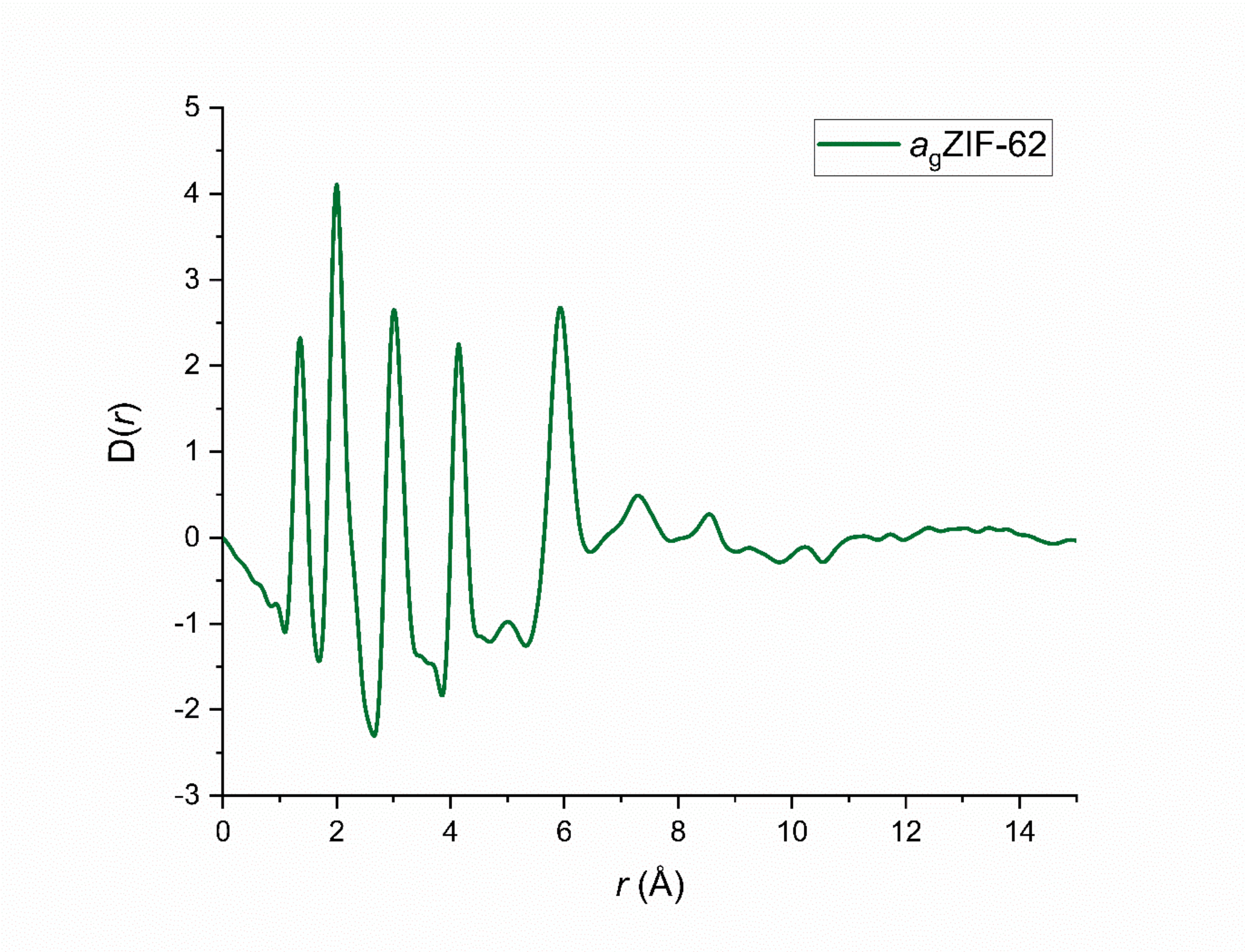

Fig. S25: D(r) for a_gZIF-62, correlations between 1 and 8 Å correspond to the local structure of the Zn-Im-Zn unit.

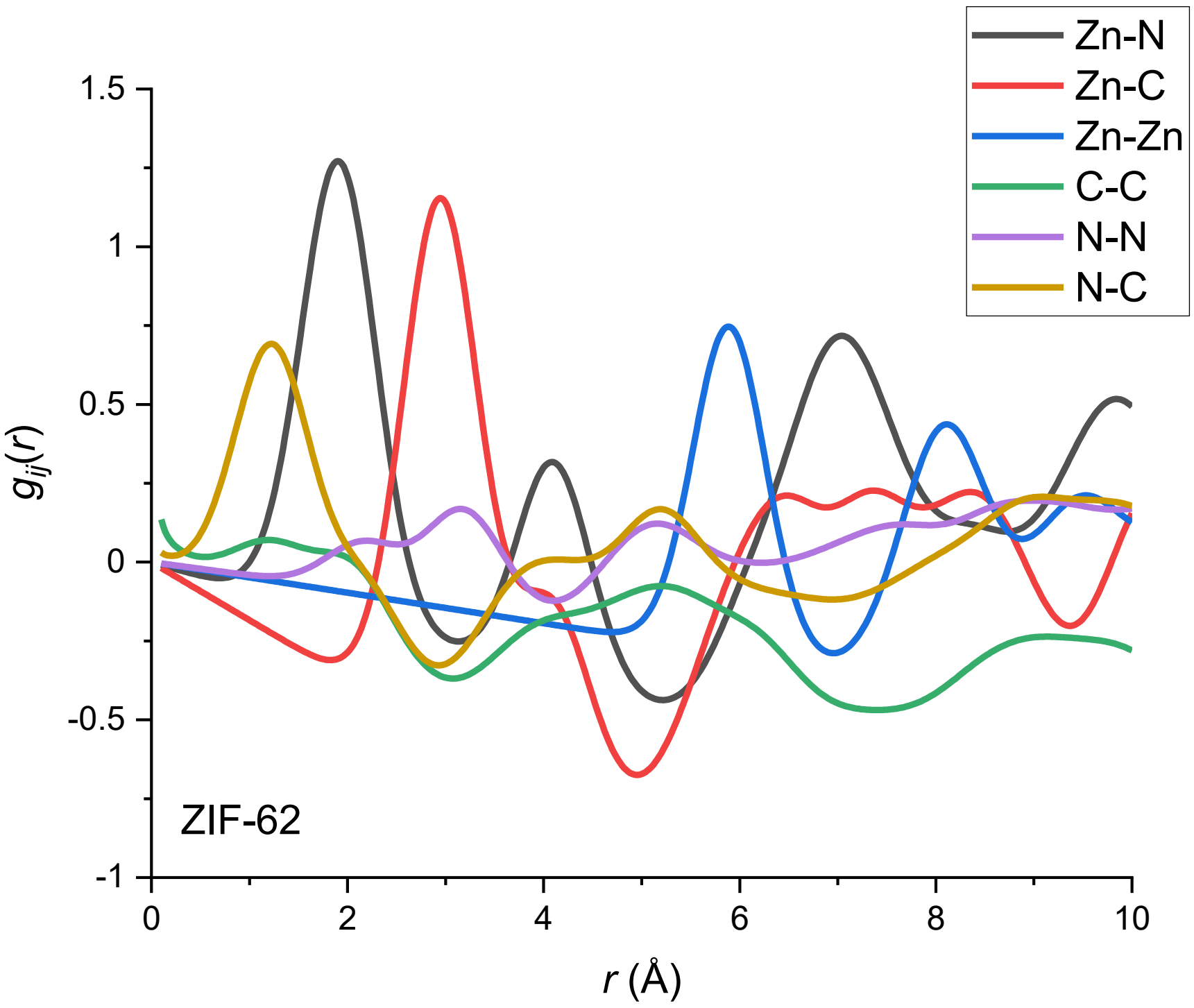

Fig. S26: gij(r) of ZIF-62 illustrating the individual correlations that contribute to the overall D(r). These gij(r) were calculated from the CIF file collected for ZIF-62.

Table S12: Partial PDF assignments for a_gZIF-62

ZIF Sample	*r* / Å	Assignment
a_gZIF-62	1.36	N-C, C-C
	2	Zn-N
	3	Zn-C
	4.1	Zn-N
	6	Zn-Zn
	7.3	Zn-N

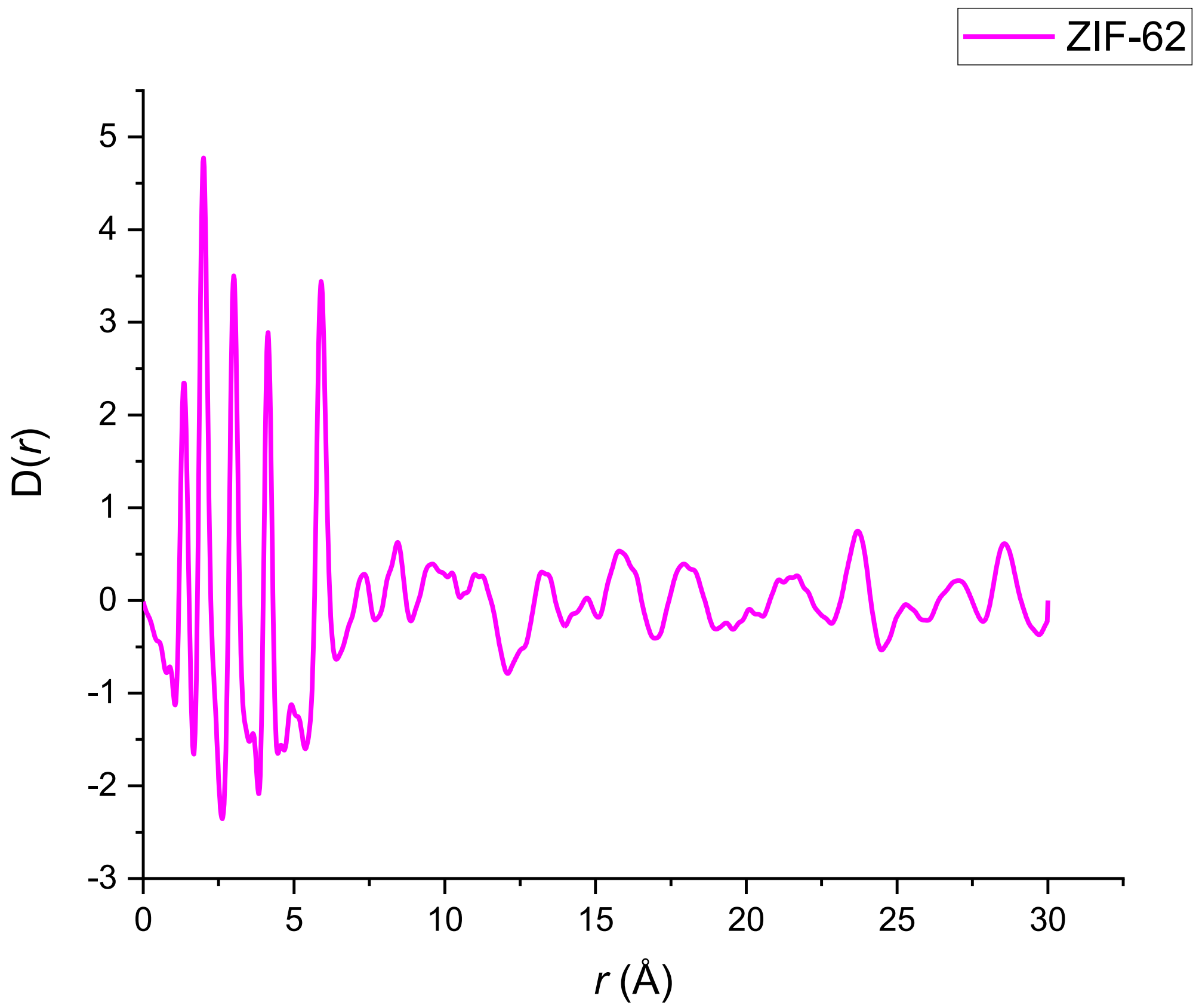

Fig. S27: D(*r*) for ZIF-62, correlations between 1 and 8 Å correspond to the local structure of the Zn-Im-Zn unit, while correlations beyond 8 Å correlate to the long-range order expected in a crystalline structure.

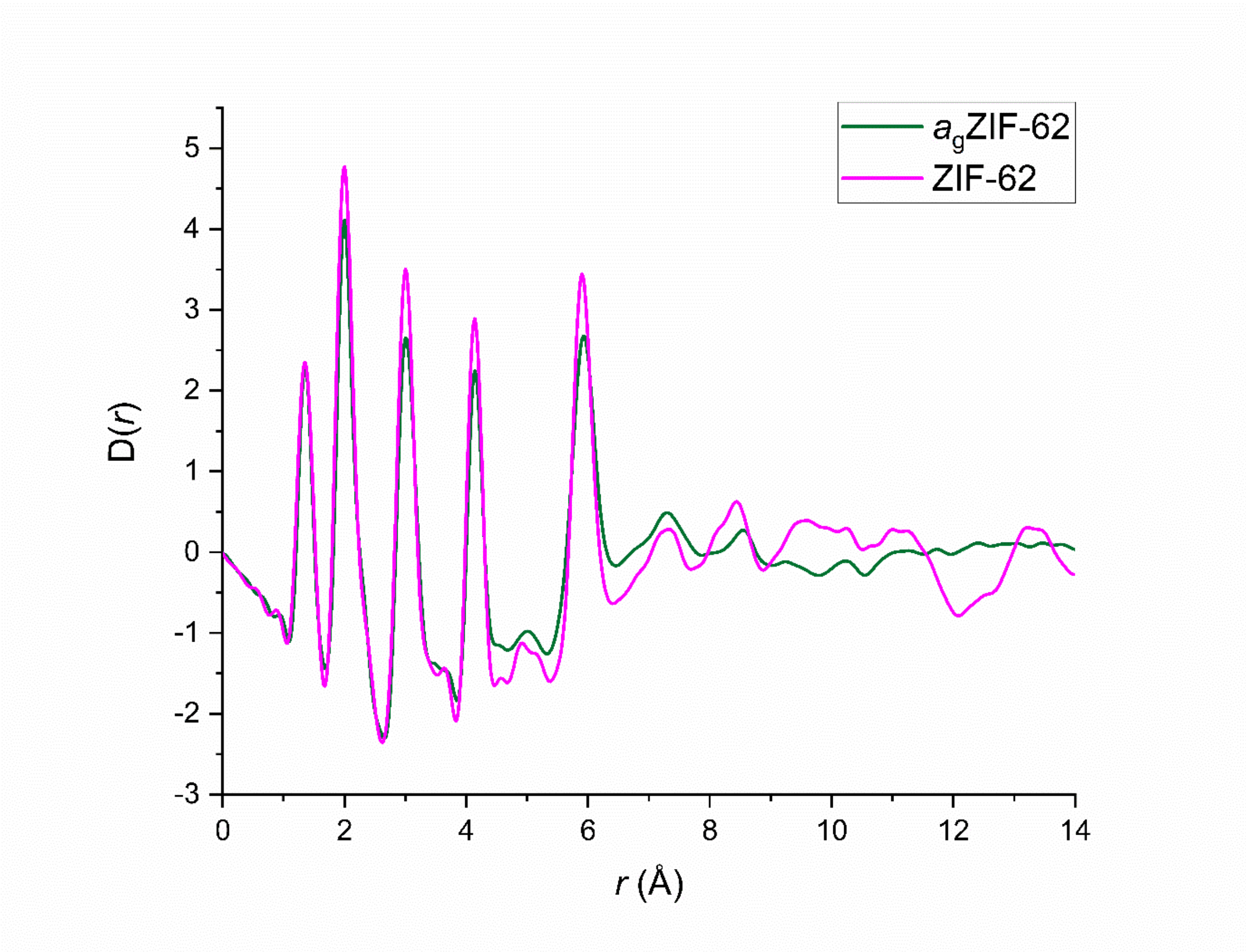

Fig. S28: D(r) for ZIF-62 and a_gZIF-62. correlations between 1 and 8 Å correspond to the local structure of the Zn-Im-Zn unit.

3.6 CO_2 Gas Sorption

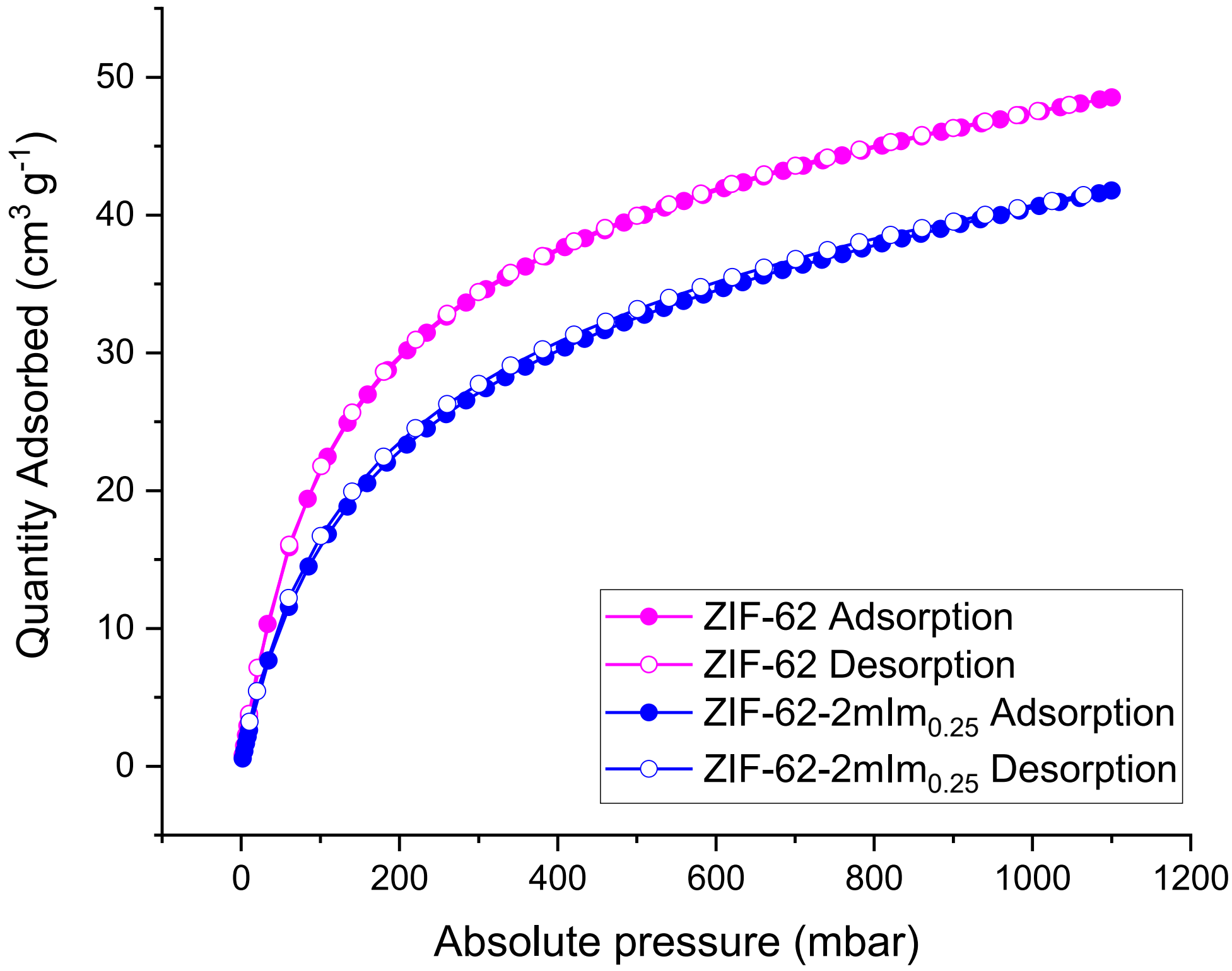

Fig. S29: CO_2 isotherms for ZIF-62 and ZIF-62-2mIm$_{0.25}$ measured at 273 K at pressures up to 1100 mbar, highlighting the difference in uptake observed for each ZIF.

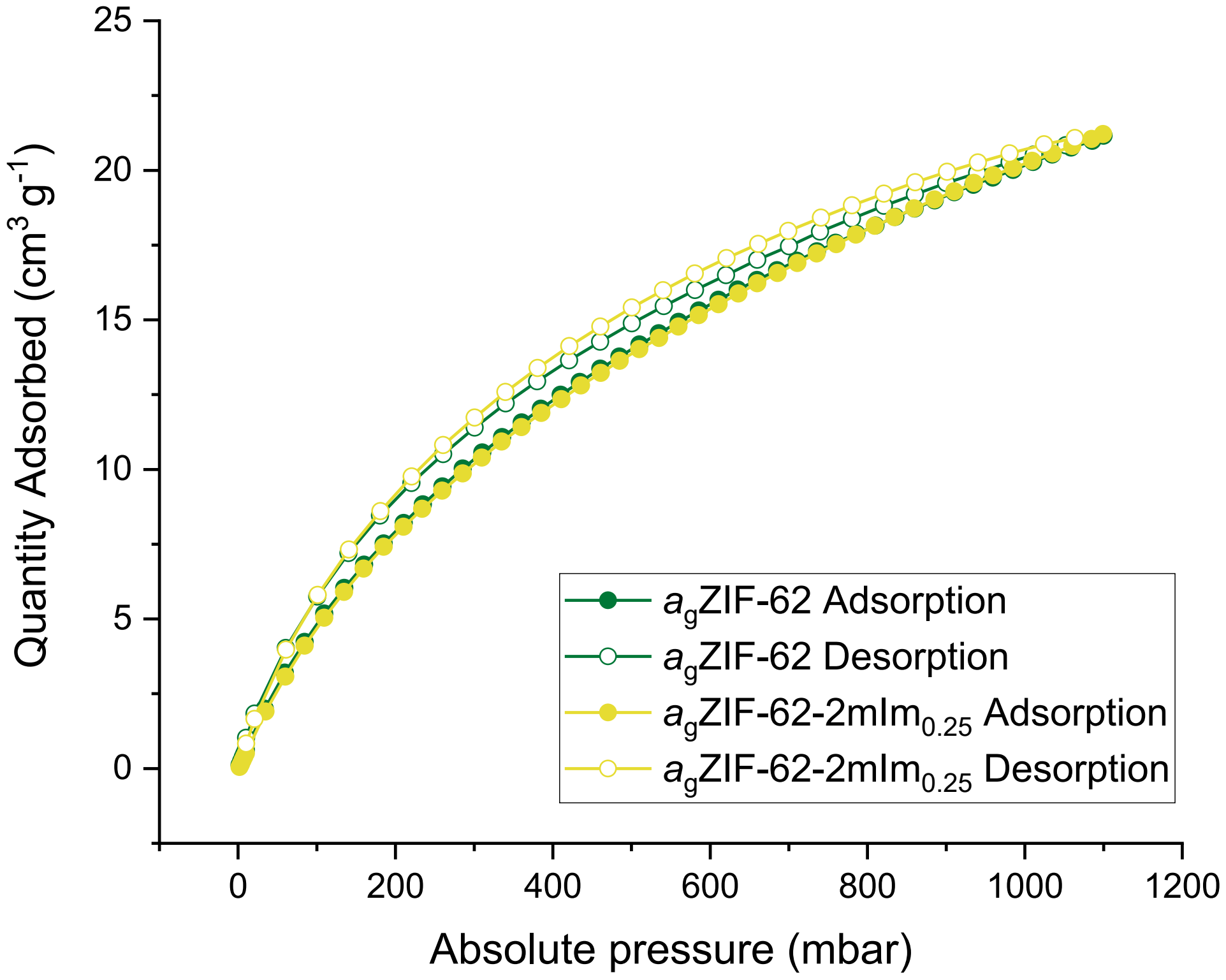

Fig. S30: CO_2 isotherms for a_gZIF-62 and a_gZIF-62-2mIm$_{0.25}$ measured at 273 K at pressures up to 1100 mbar, highlighting the similarity in uptake observed for the two ZIFs.

Table S13: Summary of maximum gas uptakes, pore volumes, and BET surface areas for CO_2 uptake measurements performed at 195 K, where the pore volumes and BET surface areas are reported only for comparison between the studied materials.

Material	Maximum uptake / cm^3 g^{-1}	Pore volume / cm^3 g^{-1}	BET surface area / m^2 g^{-1}
ZIF-62	127.7	0.20	299
a_gZIF-62	59.3	0.09	143
ZIF-62-2mIm$_{0.25}$	117.7	0.18	268
a_gZIF-62-2mIm$_{0.25}$	66.1	0.10	158

Table S14: Maximum gas uptakes for CO_2 uptake measurements performed at 273 K.

Material	Maximum uptake / cm^3 g^{-1}
ZIF-62	48.4
a_gZIF-62	21.2
ZIF-62-2mIm$_{0.25}$	41.8
a_gZIF-62-2mIm$_{0.25}$	21.5

4. References

1 R. K. Harris, E. D. Becker, S. M. C. De Menezes, P. Granger, R. E. Hoffman and K. W. Zilm, *Magn. Reson. Chem.*, 2008, **46**, 582–598.

2 C. R. Morcombe and K. W. Zilm, *J. Magn. Reson.*, 2003, **162**, 479–486.

3 B. Fung, A. Khitrin and K. Ermolaev, *J. Magn. Reson.*, 2000, **142**, 97–101.

4 A. M. Rose, A. R. McLauchlin, G. Wilson, T. O. McDonald and F. Blanc, *Polym. Chem.*, 2024, **15**, 1511–1521.

5 Netzsch, Proteus-Thermal Analysis 6.1.0 2024.

6 G. Winter, *J. Appl. Cryst.,* **43**, 2010, 186-190.

7 G. M. Sheldrick, *Acta Crystallogr A*, 2015, **71**, 3–8.

8 G. M. Sheldrick, *Acta Crystallogr C*, 2015, **71**, 3–8.

9 O. V. Dolomanov, L. J. Bourhis, R. J. Gildea, J. A. K. Howard and H. Puschmann, *J. Appl. Crystallogr.*, 2009, **42**, 339–341.

10 A. Spek, *Acta Crystallogr. Sect. C*, 2015, **71**, 9–18.

11 A. Spek, *J. Appl. Crystallogr.*, 2003, **36**, 7–13.

12 Alan Coelho, Topas Academic V7 Coelho Software 2020.

13 A. K. Soper, *Rutherford Appleton Lab. Tech. Rep.*

14 C. L. Farrow, P. Juhas, J. W. Liu, D. Bryndin, E. S. Božin, J. Bloch, T. Proffen and S. J. L. Billinge, *J. Phys. Condens. Matter*, 2007, **19**, 335219.

15 T. D. Bennett, Y. Yue, P. Li, A. Qiao, H. Tao, N. G. Greaves, T. Richards, G. I. Lampronti, S. A. Redfern, F. Blanc, O. K. Farha, J. T. Hupp, A. K. Cheetham and D. A. Keen, *J Am Chem Soc*, 2016, **138**, 3484–92.

16 A. Qiao, T. D. Bennett, H. Tao, A. Krajnc, G. Mali, C. M. Doherty, A. W. Thornton, J. C. Mauro, G. N. Greaves and Y. Yue, *Sci. Adv.*, 2018, **4**, eaao6827.

17 R. Banerjee, A. Phan, B. Wang, C. Knobler, H. Furukawa, M. O'Keeffe and O. M. Yaghi, *Science*, 2008, **319**, 939–43.
